# Additive‐Free Sequential Thermal Evaporation of Near‐Intrinsic Pb‐Sn Perovskites

**DOI:** 10.1002/smtd.202401246

**Published:** 2024-12-06

**Authors:** Lara M. van der Poll, Niels van Silfhout, Jasmeen Nespoli, Maartje van der Meer, Reinder K. Boekhoff, Lars J. Bannenberg, Arno H.M. Smets, Tom J. Savenije

**Affiliations:** ^1^ Department of Chemical Engineering Delft University of Technology Van der Maasweg 9 Delft 2629 HZ The Netherlands; ^2^ Department of Electrical Sustainable Energy Delft University of Technology Mekelweg 4 Delft 2628 CD The Netherlands; ^3^ Department of Radiation Science and Technology Delft University of Technology Mekelweg 15 Delft 2629 JB The Netherlands

**Keywords:** additive‐free, low bandgap, thermal evaporation, tin‐lead perovskites, vacuum deposition

## Abstract

To boost the efficiency of perovskite solar cells beyond the limit of a single‐junction cell, tandem cells are employed, requiring low bandgap materials. This is realized by partially substituting lead(II) (Pb^2+^) with tin(II) (Sn^2+^) in the perovskite structure. In this work, a scalable method is presented to produce formamidinium lead tin iodide (FAPb_0.5_Sn_0.5_I_3_) films by sequential thermal evaporation (sTE) of PbSnI_4_, which is an alloy of SnI_2_ and PbI_2_, and FAI, in vacuum. Annealing at 200 °C yields a highly oriented and crystalline layer comprising grains over 1 µm on average. Photoconductance measurements reveal carrier lifetimes exceeding 2 µs and mobilities ≈100 cm^2^/(Vs). Structural analysis confirms that, while interdiffusion is abundant even at room temperature, the complete conversion requires high temperatures. Although the incorporation of Cs^+^ into the A‐site of the perovskite increases the grain size, charge carrier dynamics are reduced. A comparison between the sTE films and spin‐coated samples of the same composition demonstrates the superior photoconductance of the sTE films, without the need for any additives. Overall, this study showcases the potential of sTE for producing high‐quality low band gap (LBG) perovskite materials.

## Graphical abstract

Low bandgap materials are imperative for high‐efficiency tandem solar cells. This work uses sequential thermal evaporation in vacuum to produce lead‐tin perovskites, yielding crystalline and compact films. The effects of different annealing temperatures and the incorporation of Cs^+^ into the A‐site are explored. Also, the films are compared to spin‐coated samples to demonstrate their near‐intrinsic nature without using any additives.

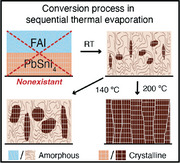

## 1 Introduction

Single‐junction (1‐j) perovskite solar cells (PSCs) nowadays achieve efficiencies comparable to conventional silicon cells.^[^ ^1^ ^]^ Even so, substantial gains in the performance of 1‐j PSCs are no longer obtainable due to energy losses caused by thermal relaxation of hot carriers and sub‐bandgap photons, described by the detailed balance limit. Using multiple absorber layers with different bandgaps in multi‐junction cells circumvents these limitations and can further boost the efficiencies of PSC modules.^[^ ^2^ ^]^ Thus far, efforts to integrate perovskites in tandem with silicon or other‐bandgap perovskites have yielded efficiencies up to 34.6% and 28.2%, respectively, for a 1 cm^2^ cell.^[^ ^3^, ^4^ ^]^

A key factor in the appeal of perovskites for photovoltaic applications is their tunable bandgap, which depends on the composition of the characteristic ABX_3_ structure. To achieve low band gap (LBG) perovskites with bandgaps down to 1.24 eV, it is necessary to partially substitute the lead(II) (Pb^2+^) with tin(II) (Sn^2+^). The use of lead‐tin (Pb‐Sn) perovskites is challenging due to the relatively low oxidation potential of Sn^2+^ to Sn^4+^. The release of electrons triggers a chain of reactions, resulting in the degradation of the perovskite lattice and, consequently, its optoelectronic properties.^[^ ^5^, ^6^ ^]^ The defects caused by the Sn^2+^ oxidation are detrimental to the PSC performance as they boost nonradiative recombination, compromising the open‐circuit voltage (V_OC_) and Fill Factor (FF).^[^ ^7^ ^]^

The low capital investment, fast iteration times, and the flexibility of solution processing make that, historically, most of the research has been focused on spin‐coated (SC) samples.^[^ ^5^, ^6^, ^7^, ^8^, ^9^, ^10^ ^]^ An increasing body of research is available for the thermal evaporation (TE) of lead‐based perovskites, focusing either on coevaporation (cTE)^[^ ^11^, ^12^, ^13^ ^]^ or sequential evaporation (sTE).^[^ ^14^, ^15^, ^16^ ^]^ TE has garnered increased attention recently, primarily because it facilitates the conformal coating of compact and uniform films over large areas and is already a well‐established method in the industry for depositing other films.^[^ ^17^ ^]^ In cTE, all precursors are deposited simultaneously. The ratios of the evaporation rates determine the stoichiometry of the resulting film. Rather, sTE is a process that occurs in distinct steps, in which each of the precursors is deposited in a layer‐by‐layer approach. This offers the advantage of being a relatively straightforward process in which the stoichiometry can be finely controlled by layer thickness. A notable drawback of sTE is the prolonged processing time required. Both TE modes have enabled the production of 1‐j Pb‐based PSCs with champion efficiencies of ≈19% and 24%, for cTE and sTE, respectively.^[^ ^11^, ^14^ ^]^

The available work on the TE of LBG perovskites entails only cTE.^[^ ^18^, ^19^, ^20^ ^]^ The films typically consist of a combination of the precursors lead(II) iodide (PbI_2_), tin(II) iodide (SnI_2_), formamidinium iodide (CH_3_(NH_2_)_2_I, FAI), methylammonium iodide (CH_3_NH_3_I, MAI) and/or cesium iodide (CsI). They are either deposited through a dual‐source method, employing an alloy of the inorganic precursors in one source and FAI in the other,^[^ ^18^ ^]^ or by a combination of multiple sources, each containing one specific precursor.^[^ ^19^, ^20^ ^]^ Additionally, the introduction of tin(II) fluoride (SnF_2_) into the alloy or as a separate source has been explored, showing that while some SnF_2_ aids the crystallization into high‐quality films, excess SnF_2_ can result in crystal phases associated with impurities or unreacted precursors. Nearly without exception, SnF_2_ is used in wet processing methods due to its significant role in mitigating the presence of Sn^4+^ and aiding the formation of oriented and crystalline films.^[^ ^8^, ^21^, ^22^ ^]^ While efficiencies nearing 14% have been achieved by incorporating cTE Pb‐Sn perovskite films in 1‐j PSCs,^[^ ^19^ ^]^ limited research has been conducted on the material properties and their optoelectronic behavior, as well as comparing them to their SC counterparts.

Commonly, both solution‐ and vacuum‐based methods apply a thermal annealing step after the deposition. This is most extensively studied for samples made from solution, where it has been associated with the removal of residual solvents, the (re)crystallization to the photoactive cubic phase, grain growth, improved morphology, and surface coverage by controlling the nucleation and growth kinetics.^[^ ^16^, ^23^ ^]^ While similar observations have been made for TE samples,^[^ ^12^, ^24^, ^25^ ^]^ the exact purpose of annealing is largely unexplored and unoptimized. cTE samples are regularly left unannealed as the precursors are sufficiently mixed during the deposition to induce the crystallization into the perovskite phase.^[^ ^11^, ^18^, ^26^, ^27^ ^]^ However, annealing procedures lasting 10 to 30 min between 100 and 170 °C have also been installed for both cTE and sTE samples to improve interdiffusion and induce (re‐)crystallization.^[^ ^12^, ^14^, ^28^, ^29^, ^30^, ^31^ ^]^ Excessively high annealing temperatures have been reported to result in re‐evaporation of the organic component and film degradation.^[^ ^24^, ^30^ ^]^

In this work, we demonstrate the sTE of LBG Pb‐Sn perovskite films. The process encompasses the in‐house production and deposition of an alloy of SnI_2_ and PbI_2_, followed by the deposition of FAI. This approach yields crystalline and compact perovskite films with a high preferential orientation in the (h00) direction. We find that high annealing temperatures are required to obtain films with carrier lifetimes exceeding 2 µs and mobilities beyond 100 cm^2^/ (Vs) using nanosecond laser pulses of 5 × 10^10^ photons/cm^2^ using microwave conductivity measurements. Moreover, it is observed that the partial replacement of FA^+^ by any amount of Cs^+^ at the A‐site of the perovskite structure limits the photoconductance. By comparing the TE perovskite films with SC samples of the same composition, the superiority of TE as a deposition method for Pb‐Sn perovskites is demonstrated, while eliminating the need for additives.

## 2 Film Fabrication and Annealing Treatment

**Figure 1:**
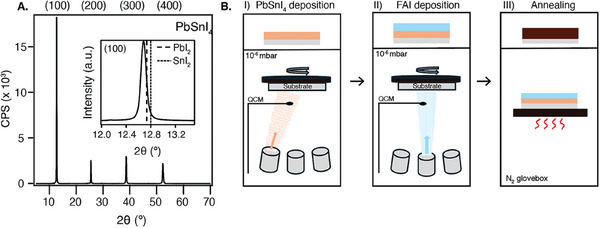
A) XRD pattern of PbSnI_4_. The inset is a zoom‐in of the (100) reflection, in which the (100) reflection of PbI_2_ (dashed line) and SnI_2_ (dotted line) are indicated. B) Schematic representation of the thermal evaporation process in which sequentially I) PbSnI_4_ (orange) and II) FAI (blue) are deposited on a quartz substrate (grey) in vacuum and III) annealed in an N_2_‐filled glovebox to form the perovskite layer (dark brown). QCM: quartz crystal microbalance.

The production of FAPb_0.5_Sn_0.5_I_3_ starts with the preparation of an alloy of PbI_2_ and SnI_2_, from here on referred to as PbSnI_4_. The alloy was synthesized in‐house by heating the mixed precursors to 420 °C under inert conditions, as detailed in the Experimental Methods. This approach was adopted to speed up the deposition and stabilize the Sn^2+^.^[^ ^5^ ^]^ The formation of the alloy was confirmed by XRD. The diffraction peaks visible in **Figure** **1**A located at 12.7°, 25.5°, 38.6°, and 52.3° correspond to the (100), (200), (300), and (400) planes.^[^ ^32^ ^]^ The inset in Figure 1A shows that the (100) reflection of the alloy is at lower diffraction angles than for PbI_2_ or SnI_2_. This indicates a larger lattice parameter, which is consistent with previous findings for mixed Pb‐Sn materials compared to pure lead or tin perovskites.^[^ ^9^ ^]^ Composition analysis via SEM EDX measurements of the evaporated PbSnI_4_ film yielded an average Pb:Sn ratio of 1:1.0 ± 0.04 and Pb:I ratio of 1:3.5 ± 0.2.

Moreover, the alloy exhibits a distinct sublimation temperature (220 °C) from PbI_2_ (245 °C) and SnI_2_ (175 °C). This indicates the formation of a single material instead of a mixture, as this would have resulted in a range of evaporation temperatures starting at 175 °C. Moreover, in the case of a mixture, the sublimation temperature would vary depending on the number of times the alloy was used, which would result in a changing composition of the final perovskite film over different depositions with the same alloy. No such observations were noted.

After the production of PbSnI_4_ was confirmed, the alloy was used for the sTE of FAPb_0.5_Sn_0.5_I_3_ (abbreviated by FA). In vacuum (10^−6^ mbar), first a layer of PbSnI_4_ was deposited onto a rotating quartz substrate, followed by FAI, as schematically depicted in Figure 1B. The evaporation rates were measured by a quartz crystal microbalance (QCM).

**Figure 2:**
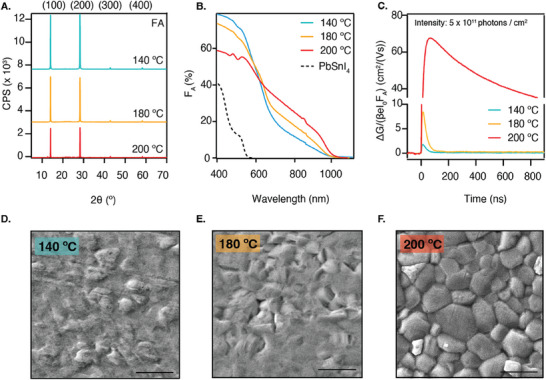
A) XRD patterns of FA films (from top to bottom) annealed at 140, 180, and 200 °C for 10 min. The Miller indices of the reflections are labeled above. B) Absorbance spectra, expressed as the fraction of absorbed light (*F_A_*) of the films annealed at different temperatures and PbSnI_4_. C) Photoconductance data as a function of time measured with excitation wavelength 800 nm with an intensity of 5 × 10^11^ photons/cm^2^. The traces are normalized by the laser intensity (*I*_0_) and *F_A_*. Top‐view SEM images of the films annealed at D) 140 °C, E) 180 °C, and F) 200 °C. The scale bar represents 1 µm.

After the deposition, the samples were transferred from the evaporation chamber to a N_2_‐filled glovebox, where some were annealed in series on a hot plate for 10 min at annealing temperatures (*T_anneal_*) of 140, 180, or 200 °C. This resulted in 145 nm thick layers. **Figure** **2**A shows that independent of *T_anneal_*, only the (100), (200), (300), and (400) reflections are visible at 14.0°, 28.2°, 42.8°, and 59.3°, showing a highly preferential orientation akin to the alloy film. The tendency of directional growth exists in thermally evaporated perovskites, both made by sTE and cTE,^[^ ^13^, ^20^, ^30^ ^]^ more so than in solution‐processed samples. This directional growth is likely dictated by the structure of the substrate or, in the case of sTE, the first layer deposited on a specific substrate, which acts as a seed layer for a certain orientation.^[^ ^30^ ^]^ The growth direction of the seed layer can then propagate throughout the entire film. In contrast, crystallization in solution‐processed films typically occurs due to the rapid precipitation induced by an antisolvent and subsequent isotropic growth, resulting in randomly oriented crystal domains.^[^ ^21^, ^33^ ^]^ The degree of preferential orientation along certain crystal planes has been shown to enhance the crystallinity and the charge transport properties by reducing the number of grain boundaries. Besides all samples showing preferential orientation, a more prominent peak is present at 12.7° for the sample annealed at 200 °C, which is attributed to PbSnI_4_ (see Figure S1A, Supporting Information), indicating that the sample has started to revert into the precursors.

Additionally, the thickness of the films decreases by ≈30 nm with increasing annealing temperature (see Figure S1B, Supporting Information). We exclude that a significant decrease in the lattice parameter contributes to this finding, as it only exhibits a minor decrease of 0.007 Å compared to the unannealed sample (see Figure S1C, Supporting Information). Instead, the lower thickness is related to the desorption of excess organic precursor, which is confirmed by SEM EDX (see inset in Figure S1B, Supporting Information). We observe a decrease in the elemental contribution of nitrogen (N) and carbon (C), while the lead, tin, and iodide contributions are virtually unchanged. This shows that this thickness reduction correlates with a decrease in FA^+^ (CH_3_(NH_2_)_2_^+^) content within our sample.

The bandgap was determined to be 1.28 eV (see Tauc plot in Figure S1D, Supporting Information), which is in agreement with previously reported values for this composition.^[^ ^9^, ^34^ ^]^ Furthermore, the absorbance measurements, presented in Figure 2B, show that the higher *T_anneal_* results in a more distinct absorption onset and a larger fraction of absorbed light (F_A_) at wavelengths beyond 600 nm. This suggests that higher annealing temperatures enhance the conversion into the perovskite phase. Moreover, the absorbance ≈500 nm progressively reduces upon annealing, which can be seen from the absorption shoulder which is clearly visible in the sample annealed at 140 °C and already less pronounced in the sample annealed at 180 °C. This shoulder corresponds to the absorption of the alloy, which is included in Figure 2B, and suggests that there are still unreacted precursors, although not visible from XRD. At 200 °C, this shoulder has completely disappeared, and different absorption features arise. These features have been previously described to occur in thermally evaporated FA^+^‐rich Pb^2+^‐perovskites and are attributed to the presence of electronically confined nano‐structured domains of size 10–20 nm.^[^ ^35^, ^36^ ^]^

To study the impact of the annealing temperature on the charge carrier dynamics, time‐resolved microwave conductivity (TRMC) measurements were performed. Figure 2C shows the photoconductance (Δ*G*) signals as a function of time for each annealing temperature. The traces are normalized by a geometric factor (β), the electronic constant (*e*), the laser intensity (*I_o_*), and *F_A_*, which allows us to express the data as a yield‐mobility product. At *t* = 0, a laser pulse strikes the sample, generating mobile charges. Assuming constant hole and electron mobilities over time, these charges recombine or are immobilized by traps. This means that they no longer contribute to the TRMC signal, which appears as a decay over time. In the context of TRMC, the carrier lifetime is defined as the time that it takes for the Δ*G* signal to reduce to half of its initial height. We observe in Figure 2C that raising the annealing temperature enhances the photoconductance signal. Moving from annealing at 140 to 180 °C, the mobility of the generated carriers improves from 1.5 cm^2^/(Vs) to 5 cm^2^/(Vs) when employing a light intensity of 5 × 10^11^ photons/cm^2^. In this scenario, the carrier lifetime remains 38 ns. A substantial jump in Δ*G* is induced when further increasing the temperature to 200 °C, resulting in much higher mobilities higher than 50 cm^2^/(Vs) and elongated carrier lifetimes of 2.5 µs, which is better visualized by the longer timescale presented in Figure S2D (Supporting Information).

To enable a more comprehensive investigation of the carrier dynamics, TRMC measurements are usually performed across a range of laser intensities to generate different light‐induced carrier concentrations, as presented in Figure S2A–C (Supporting Information). The TRMC signals of the traces annealed at 140 and 180 °C decay so fast that within the response time of the cavity cell (18 ns, see information in Experimental Methods), most of the charges have already recombined. In other words, the lifetime of the generated charges lies somewhere within the pulse width and the measured data for those samples represents the very tail of the decay signal due to a very fast and dominant immobilization and/or recombination mechanism. The source of this phenomenon seems to be removed upon annealing at 200 °C and long lifetimes and high mobilities are obtained. In Figure S2C (Supporting Information) we can see the altogether lowering of the TRMC signal with increasing light intensities, indicating that a second‐order process is dominating the carrier recombination dynamics.

The enhancement of the photoconductance is observed together with a relief in microstrain in the out‐of‐plane direction of the thin film. An unstrained, densely packed, and crystalline perovskite film is better able to retain charges without inducing nonradiative recombination.^[^ ^37^, ^38^ ^]^ This notion was studied by comparing the widths (FWHM) of the available XRD peaks between different annealing temperatures (Figure S1E, Supporting Information) (see procedure in Experimental Methods). Since only the (h00) reflections are visible, we can only deduce the microstrain along that specific direction. The slopes of the resulting plots were used to determine the corresponding reduction in microstrain, which decreases from 0.03% to 0.0002% with higher *T_anneal_* (Figure S1F, Supporting Information).

Lastly, the SEM pictures presented in Figure 2D–F show that annealing at 140 °C results in a smooth film with an amorphous surface. The amorphous phase starts to disappear upon annealing at 180 °C and a complete grain structure appears only when annealing at 200 °C, resulting in a film with grains up to 1 µm in size mixed with smaller grains. Lower magnifications included in Figure S3 (Supporting Information) show that all films homogeneously and compactly cover the entire substrate surface, which is a characteristic of thermally evaporated samples.

All in all, a high *T_anneal_* is required to produce a semiconducting material with high photoconductance produced by sTE. A prerequisite in forming perovskite through this method is sufficient mixing of the different layers of precursors by interdiffusion. To test the extent to which the PbSnI_4_ and FAI mix at different applied *T_anneal_*, we performed XPS measurements with depth profiling on samples annealed at 200 and 140 °C (Figure S4A,B, Supporting Information). The XPS survey scans are included in Figure S5 (Supporting Information). Additionally, a sample that was left unannealed was analyzed (Figure S4C, Supporting Information), for which the XRD, absorption, and TRMC data are included in Figure S6 (Supporting Information). Interestingly, aside from the surface region represented by the first 20 s of etching, all films have a constant composition throughout the film's thickness. Moreover, to test the uniformity of the crystal structure in the direction perpendicular to the precursor layers, we performed grazing‐incidence XRD (GIXRD) with different incidence angles (0.5°, 1°, 3°, and 5°) for samples annealed at 140 and 200 °C. The results (Figure S7, Supporting Information) evidence that both samples show no pronounced incident angle dependences, indicating that at different depths of the film, the structure and composition of the perovskite phase are the same.

Finally, cross‐sectional SEM images (Figure S8, Supporting Information) reveal that the samples produced by sTE consist of a continuous material in the thickness of the film. Similar to the top‐view images, the grain structure is significantly more visible in the sample annealed at 200 °C than in the sample annealed at 140 °C. However, some voids and pinholes are visible in the film annealed at 200 °C that are not visible from the top view.

**Figure 3:**
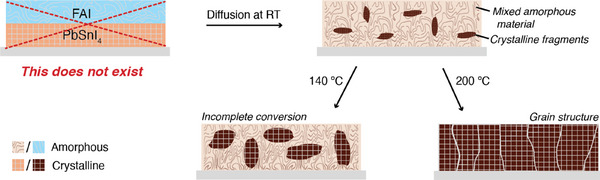
Diagram portraying the proposed conversion mechanism of precursors into the perovskite crystal structure in the process of sequential thermal evaporation.

The observations discussed in this section lead us to the following notion of the actual conversion of the precursor layers into perovskite in the process of sequential thermal evaporation, schematically illustrated in the diagram in **Figure** **3**. The first step entails the actual thermal evaporation of precursor layers. Most importantly, we see that ions diffuse and form an amorphous mixed material, even at room temperature (Figure S4C, Supporting Information). Upon introduction of FAI, there is no sign of PbSnI_4_ in the XRD pattern of the unannealed film (Figure S6A, Supporting Information), indicating the formation of a mixed amorphous material. Differently, characteristic perovskite peaks are already present in the XRD pattern. This implies that the amorphous precursors coexist with a perovskite structure and the formation of perovskite can occur even without any annealing. It also implies that ion migration is abundant and not limiting the conversion, which is further supported by the XPS results. Depending on the annealing temperature, more material is converted into the perovskite structure, as the absorbance between 600–1000 nm clearly increases with increasing temperature (Figure 2B). We anticipate that at 200 °C most of the amorphous mixed material is converted, although due to the evaporation of FAI a nonstoiciometric mixture is formed at the surface leading to the formation of some PbSnI_4_ (Figure S1A, Supporting Information). This indicates that conversion from the amorphous mixed precursors into perovskite is temperature‐activated and that higher temperatures convert a larger fraction of the bulk amorphous material into perovskite. Only once a major part of the bulk is converted decent charge carrier lifetimes are observed. This implies that grains embedded in the amorphous mixed system suffer from defects states at the grain boundaries leading to short carrier lifetimes.

## 3 A‐Site Engineering: Cs^+^ Addition

To further optimize the perovskite's optoelectronic properties, the occupancy of the A‐site in the perovskite structure has been studied extensively over the past years.^[^ ^9^, ^39^, ^40^, ^41^ ^]^ Especially the ionic radii of the A‐site cations are relevant for the structural stability of the radiative cubic phase. Cations that are too small cause the BX_6_ octahedral structure to tilt, resulting in orthorhombic structures that are not photoactive. Yet, excessively large cations deform the structure by stretching out the lattice.^[^ ^9^, ^42^ ^]^ This explains why the commonly used, but large, methylammonium (MA^+^, ionic radius: 2.16 Å) and formamidinium (FA^+^_,_ ionic radius: 2.53 Å) are partially substituted with cesium (Cs^+^, ionic radius: 1.67 Å) in the highest‐performing PSCs.^[^ ^39^, ^43^ ^]^ Moreover, the addition of Cs^+^ has been shown to influence the crystallization process from solution due to differences in the coordination chemistry compared to the organic MA^+^ and FA^+^.^[^ ^44^ ^]^

We explored the incorporation of Cs^+^ into the A‐site of the thermally evaporated LBG perovskites as well. This is achieved by adding a third step into the deposition process in which a layer of CsI is deposited on top of the FAI layer (see Figure S9, Supporting Information). In this manner, perovskite films of composition Cs_x_FA_1‐x_Pb_0.5_Sn_0.5_I_3_ with x = 0.05, x = 0.25, and x = 0.40 were prepared, respectively referred to as Cs_5_FA_95_, Cs_25_FA_75_, and Cs_40_FA_60_. This approach yielded layers of ≈140–150 nm thick (Table S1, Supporting Information). The annealing series presented in Figure 2 was repeated for Cs_5_FA_95_ (see Figure S10, Supporting Information) which showed similar trends to FA. Thus, all samples were annealed at 200 °C for 10 min after the deposition.

**Figure 4:**
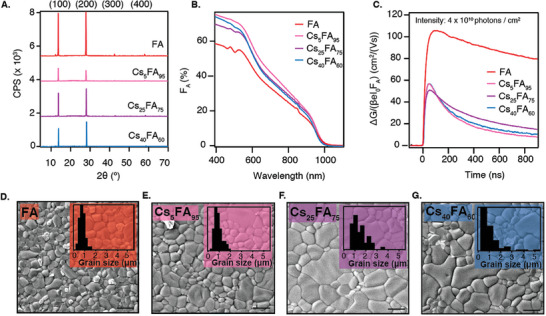
A) Normalized XRD pattern of (from top to bottom) FA, Cs_5_FA_95_, Cs_25_FA_75_, and Cs_40_FA_60_ films. The Miller indices of the reflections are labeled above. B) Absorbance spectra expressed as F_A_. C) Photoconductance data as a function of time measured with excitation wavelength 800 nm with an intensity of 4 × 10^10^ photons/cm^2^. The traces are normalized by *I*_0_ and *F_A_*. Top‐view SEM images of D) FA, E) Cs_5_FA_95_, F) Cs_25_FA_75_, and G) Cs_40_FA_60_ films. The scale bar represents 1 µm. The bar plots in the inset show the grain size distribution.

**Figure** **4**A shows the XRD patterns of these films plotted together with FA. Consistent with the XRD pattern of FA, the Cs^+^‐containing films show a preferential orientation in the (h00) direction. The desired composition of the Cs^+^‐containing films was confirmed by SEM EDX (see Table S1, Supporting Information). This was further validated by the shrinking lattice parameter due to the smaller ionic radius of Cs^+^ (see Figure S11A, Supporting Information). To address how well the Cs^+^ is distributed along the sample thickness, we performed XPS measurements at different depths of the film by etching (Figure S4D,E, Supporting Information). Aside from the surface region represented by the first 20 s of etching, the films have a constant composition throughout the film's thickness. These observations convince us that Cs^+^ is homogeneously distributed and incorporated into the lattice.

From the absorbance spectra in Figure 4B, it is seen that the absorption features that are apparent in FA ≈500 nm are removed upon the addition of Cs^+^, which was previously described in literature and attributed to the removal of the crystal domains that cause electronic confinement.^[^ ^45^, ^46^ ^]^ Although there are some minor differences in the absorption, the bandgap of Cs_5_FA_95_ and Cs_25_FA_75_ is unaffected compared to FA. The incorporation of a high Cs^+^ content, like in Cs_40_FA_60_, widens the bandgap to 1.29 eV (see Tauc plot in Figure S11B, Supporting Information).

TRMC was used to investigate the impact of Cs^+^ on the optoelectronic performance of the perovskite films. Figure 4C shows that FA has the highest photoconductance at an intensity of 4 × 10^10^ photons/cm^2^, with mobilities exceeding 100 cm^2^/(Vs), while the introduction of any amount of Cs^+^ nearly halves the maximum Δ*G* signal, resulting in mobilities of 50 – 60 cm^2^/(Vs). The carrier lifetime decreases from 2.5 µs (see Figure S2D, Supporting Information) to below 500 ns for all Cs^+^ concentrations. Furthermore, we use TRMC to measure the Urbach energy (*E_U_*) to assess the electronic disorder in the samples. The results, included in Figure S13 (Supporting Information), show that the Urbach energies are comparable, 17.4 ± 0.8 and 16.6 ± 0.4 meV for FA and Cs_40_FA_60_, respectively.^[^ ^47^, ^48^ ^]^

Surprisingly, the detrimental effect of Cs^+^ on the photoconductance is accompanied by increasing grain sizes in the SEM images presented in Figure 4D–G. Other magnifications are included in Figure S14 (Supporting Information). This growth is coupled with a widening of the grain size distribution as shown in the insets of Figure 4D–G. The FA and Cs_5_FA_95_ films predominantly have grains of 1 µm in size, and no grains larger than 2 µm. In contrast, Cs_25_FA_75_ and Cs_40_FA_60_ also contain some larger grains, ranging between 2 – 5.5 µm. The appearance of these larger grains is attributed to the slower crystallization process in the presence of Cs^+^. To explore the structural variations between the samples with and without Cs^+^, we repeat the annealing series that was performed on FA as well as the microstrain analysis. Figures S10 and S11 (Supporting Information) show that the results do not significantly differ due to the addition of Cs^+^. This suggests that the Cs^+^‐containing samples are structurally similar to FA.

However, as indicated by the TRMC results, the addition of Cs^+^ has a negative effect on the optoelectronics. We expect that longer or higher annealing temperatures are required to suppress defects, likely located at the grain boundaries. However, this will lead to FAI evaporation compromising the structural properties. Alternatively, the mobility of the charge carriers in the Cs^+^‐containing perovskites might be lower than in the Cs^+^‐free perovskite.

These findings contradict what is observed in spin‐coated perovskites, where moderate amounts of Cs^+^ doping (≤25%) have been associated with enhanced performance.^[^ ^9^, ^10^, ^49^ ^]^ This is mostly related to the lowering of the crystallization rate, resulting in more crystalline, defect‐free films with smaller^[^ ^10^ ^]^ or unchanged^[^ ^9^ ^]^ grains and improved thermal stability of the photoactive α‐phase.^[^ ^40^ ^]^ Nonetheless, it has been reported that a Cs^+^ excess (>25%) results in inhomogeneous films with fused grains and reduced stability.^[^ ^7^, ^34^, ^41^ ^]^ Contrarily, we observed that the addition of different amounts of Cs^+^ always appears to be detrimental to the optoelectronic properties of sTE Pb‐Sn perovskite films. However, the high photoconductance of the fully FA^+^‐based perovskite suggests that Cs^+^‐addition might not be necessary to produce high‐quality LBG perovskites for use in PSCs by TE.

## 4 Additive‐Free Perovskite Films Through sTE: Comparison to cTE and SC and the SnF_2_ Effect

**Figure 5:**
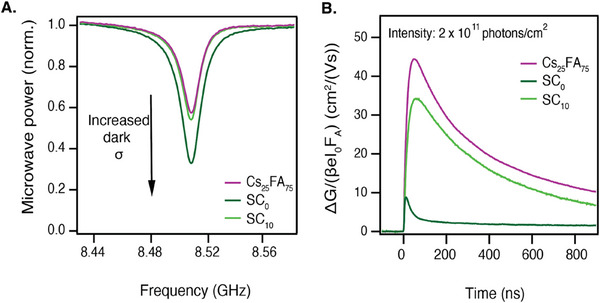
A) Steady–state microwave conductivity (SSMC) data relating the normalized microwave power to the microwave frequency for the thermally evaporated sample Cs_25_FA_75_ and spin‐coated samples of the same composition without (SC_0_) and with (SC_10_) 10% SnF_2_. B) TRMC data, measured with excitation wavelength 800 nm with an intensity of 3 × 10^11^ photons/cm^2^. The traces are normalized by *I*_0_ and *F_A_*.

Finally, it is important to compare the TE films with solution‐processed samples. Especially SC samples have been used extensively to study the properties of perovskite films. In **Figure** **5**, the TE Cs_25_FA_75_ is compared to samples of the same composition made by spin‐coating. A sample without additives (SC_0_) is included as well as a sample containing 10% SnF_2_ (SC_10_). These samples were prepared by the anti‐solvent method and subsequent annealing at 100 °C, following the procedure detailed in the Experimental Methods.

We use steady‐state microwave conductivity (SSMC) measurements to gain insight into the dark conductivity of the perovskite samples. The SSMC method probes the fraction of microwaves that is absorbed by the sample in the dark, which correlates to the background carrier concentration, as detailed in the Supporting Information. Figure 5A shows that SC_0_ has the deepest dip, corresponding to the highest dark conductivity. When SnF_2_ is added, the dip shallows, demonstrating the additive's effectiveness in mitigating the presence of background charges. The dips of SC_10_ and TE Cs_25_FA_75_ are nearly identical, and both imply near‐intrinsic semiconductor behavior.^[^ ^50^ ^]^

The TRMC traces presented in Figure 5B further highlight the thermally evaporated film's superiority in photoconductance. Other intensities for the SC samples are included in Figure S15 (Supporting Information). Upon excitation, the SC_0_ shows a very low photoconductance signal which decays within a few nanoseconds, indicating fast recombination and/or trapping of the generated carriers. The additive‐supported SC_10_ portrays an improved photoconductance signal, with longer carrier lifetimes of 350 ns and mobilities over 34 cm^2^/(Vs). Still, the TE Cs_25_FA_75_ shows the highest photoconductance with mobilities of 44 cm^2^/(Vs) and similar lifetimes to the SC_10_.

A large part of the explanation for the relative ease of producing near‐intrinsic Pb‐Sn films in vacuum is the obvious absence of solvents. Solvents, such as the frequently used dimethyl sulfoxide (DMSO) or dimethyl formamide (DMF), are never fully dry nor free of impurities. Without the presence of SnF_2_, the concentration of Sn^4+^ already increases while the precursors are still in solution.^[^ ^51^ ^]^ Moreover, the presence of residual oxygen in the glovebox during the deposition process induces Sn^2+^ oxidation.

To further study the influence of SnF_2_, we replaced 10% of the SnI_2_ with SnF_2_ before alloying with PbI_2_. We used this SnF_2_/PbI_2_/SnI_2_ alloy to produce FA films by the same procedure as before. The results show basically no changes in the XRD patterns (Figure S16A, Supporting Information). Also, the optical absorption spectra (Figure S16B, Supporting Information) are very similar. Furthermore, the films containing SnF_2_ have a similar surface grain structure as the films without SnF_2_, and in addition, the same excellent coverage, as can be seen from the top‐view SEM images (Figure S16C,D, Supporting Information). Key to note is that adding the SnF_2_ does not lead to a reduced background conductivity as deduced from Figure S16E (Supporting Information). Instead, we notice a reduction in the photoconductance signals which could be explained by a reduction of the mobility to ≈55 cm^2^/(Vs) (Figure S16F, Supporting Information). Also, the lifetimes lower to ≈500 ns. In conclusion, while the introduction of SnF_2_ does not significantly alter the structural properties of the films, it leads to a slight decline in their electronic performance. Overall, the use of SnF_2_ is redundant for the production of near‐intrinsic Pb‐Sn perovskites through sTE.

In addition, a comparison between the LBG films produced by sTE and previously reported data on LBG films made by cTE^[^ ^18^, ^19^, ^20^ ^]^ is warranted. Similarly to the films presented in this paper, the films produced by cTE show some degree of preferential orientation in the (h00) direction. Strikingly, the XRD pattern of the material produced by Ball et al.^[^ ^18^ ^]^ shows a significant contribution of the amorphous substrate, while the film is significantly thicker (400‐500 nm) than the films reported in our study (<200 nm), suggesting a lower degree of crystallinity. Furthermore, the SEM images reveal that the grain sizes in the cTE films are much smaller (10–300 nm) than those in sTE films, which are 1 µm on average. The lack of annealing of the cTE films can explain this difference in crystallinity and grain size. In terms of optoelectronic properties, Ball et al. report mobilities of 15 cm^2^/(Vs) and lifetimes below 10 ns, as measured by THz spectroscopy. These reported values are notably lower than the mobilities and lifetimes included in this work for the sTE perovskites. Overall, it seems like the films produced by these evaporation modes vary structurally and optoelectronically. Further systematic research must be done to determine how these differences affect the photovoltaic performance of these materials.

## 5 Conclusion

In conclusion, we report a scalable method of producing low bandgap Pb‐Sn perovskites by thermal evaporation. This process yields crystalline uniform, and densely packed films with low background charge concentrations, exhibiting carrier lifetimes and mobilities exceeding 2 µs and 100 cm^2^/(Vs). These films are produced by sequentially depositing the required precursors and subsequently annealing at 200 °C for 10 min. The improved properties obtained at high annealing temperatures were attributed to simultaneous FAI desorption and temperature‐activated conversion of the perovskite crystal structure. Furthermore, we demonstrate that the addition of Cs^+^ inhibits the photoconductance by reducing the lifetimes and mobilities below 500 ns and 60 cm^2^/(Vs), which is explained by a lowering of the mobility or the presence of defect‐rich grain boundaries, as the Cs^+^‐containing films are structurally similar to FA. By comparing the thermally evaporated films with spin‐coated samples of the same composition, the superiority of the former in terms of photoconductance is confirmed, eliminating the need for the additives required to stabilize solution‐processed samples. This work advances the production of near‐intrinsic low bandgap materials, which is imperative in the production of high‐efficiency solar cell configurations, by use of an industrially scalable fabrication method.

## 6 Experimental Section

### PbSnI_4_ Alloy Preparation

Tin(II) iodide beads (SnI_2_, 99.999% from beads) were ground inside a nitrogen‐filled glovebox and mixed with equimolar amounts of lead(II) iodide (PbI_2_, 99.99%). The crucible was placed inside the evaporation chamber, which subsequently was flushed three times with N_2_. After the last cycle, the chamber was filled with N_2_. The crucible was heated to 420 °C for 20 min. The resulting alloy, denoted as PbSnI_4_, was used in the perovskite deposition without any further treatment.

### Sequential Thermal Evaporation of Perovskite Layers

Quartz substrates were subsequently washed with acetone and isopropanol. The substrates were treated with oxygen plasma before they were loaded into the evaporator. In the evaporation chamber, the substrates were shortly heated to 40 °C in vacuum (10^−7^–10^−6^ mbar) to remove residual adherents. During the deposition, the substrate was continuously rotating at 40 rpm. For the synthesis of Cs_X_FA_1‐X_Pb_0.5_Sn_0.5_I_3_, sequential layers of PbSnI4, formamidinium iodide (FAI, 99.99%) and (if required) cesium iodide (CsI, 99.9%) were deposited. The crucibles containing the precursors were loaded from the N_2_ glovebox (O_2_ and H_2_O concentration < 0.1 ppm) into the evaporation chamber the day before deposition in ambient air. **Table** **1** lists the thickness of each layer, measured by a quartz crystal microbalance (QCM). The QCM is located halfway between the evaporation sources and the substrate holder. To estimate the actual deposited thickness, a tooling factor (TF) was required, also reported in Table 1. The TF is determined by equation S1, comparing the measured thickness (*d_QCM_*) with the actual thickness (*d_quartz_*) which was measured by stylus profilometry or in the case of FAI, an iterative procedure based on the XRD patterns. After the separate layers were sequentially deposited, the samples were brought into the glovebox using an air‐tight transfer unit for annealing.

(S1) $$TF=\frac{d_{quartz}}{d_{QCM}}$$

**Table 1:** Thickness, as measured in QCM, of each layer for the difference compositions. These are already corrected for the tooling factor (TF).

Sample	Thickness [nm]		
	PbSnI_4_ [TF = 0.78]	FAI [TF = 0.48]	CsI [TF = 0.72]
FA	162.5	272.1	‐
Cs_5_FA_95_	162.5	258.5	5.5
Cs_25_FA_75_	162.5	194.2	31.5
Cs_40_FA_60_	162.5	151.3	54.8

### Synthesis of Solution‐Processed Samples

Quartz substrates were subsequently washed with acetone and isopropanol in the ultrasonic bath. The substrates were dried and wiped with an antistatic wipe, after which they were placed in the UV‐ozone cleaner for 10 min. In a N_2_‐filled glovebox (O_2_ < 0.5 ppm, H_2_o < 0.8 ppm), 1.5 M parent solutions of Cs_0.25_FA_0.75_PbI_3_ and Cs_0.25_FA_0.75_SnI_3_ were prepared by mixing the required amounts of CsI, FAI, PbI_2_ and/or SnI_2_ in a 4:1 DMF:DMSO solvent mixture. The precursors that were used for the SC samples were the same as those used in TE. When 10 mol% tin(II) fluoride (SnF_2_, 99%) was added, this was done relative to the amount of SnI_2_. These mixtures were left to stir overnight. An hour before the deposition, the parent solutions were mixed and left to stir. The films were deposited using the antisolvent spin‐coating method. The rotational speed ramp was 500 rpm and the final speed 3000 rpm. 50 s after the initial deposition, 200 µL of antisolvent (anisole) was applied. Lastly, the films were annealed at 100 °C for 10 min.

### Structural Characterization

X‐ray diffraction (XRD) was done on a Bruker D8 Advance‐ECO diffractometer in Bragg‐Brentano configuration in ambient conditions. Cu‐Kα (*λ* = 1.54 Å) radiation was used. The (100), (200), (300), and (400) peaks, located at 14.1°, 28.4°, 43.1°, and 58.7°, respectively, were fitted with two pseudo‐Voigt functions to account for the presence of Kα2 radiation. The intensity ratio and position of these two pseudo‐Voigt functions were fixed according to their theoretically expected ratios.

The lattice parameter (*a*) was calculated by use of Equation S2, in which the known wavelength of the Cu‐Kα source (λ) and the miller indices of the lattice planes (*hkl*) are used together with the exact reflection angle (θ) obtained from the fits.

(S2) $$a=\frac{\lambda \sqrt{h^{2}+k^{2}+l^{2}}}{2sin\theta }$$

The microstrain in the perovskite films was evaluated by a Williamson‐Hall (W‐H) analysis. The full width at half‐maximum (FWHM) was then used to plot sinθ against FWHM*cosθ. Subsequently, linear fits were applied to extract the microstrain (ε) from the slope, following equation S3. Here, *K* is a shape factor (0.9 when a spherical crystallite shape is assumed), λ is the wavelength of the X‐ray radiation and D is the crystallite size.

(S3) $$FWHMcos\theta =4\varepsilon \sin\theta +\frac{K\lambda }{D}$$

Grazing incident X‐ray diffraction (GIXRD) measurements were performed on a Bruker D8 Discover X‐ray diffractometer in vacuum (pressure < 10^−4^ mbar) using an Anton Paar XRK900 Reactor chamber. The measurements were done with Cu‐Kα (*λ* = 1.54 Å) radiation and a LYNXEYE XE detector operated in 0D mode. On the primary side, an exit slit of 0.1, 0.2, 0.6 and 1.0 mm was used for incident angles 0.5°, 1°, 3° , and 5°, respectively. On the secondary side, a 0.6° Soller slit was used to control the beam divergence. The information depth was calculated using the mass attenuation coefficient of Pb (166 cm^2^ g^−1^), Sn (166 cm^2^ g^−1^), I (198 cm^2^ g^−1^), C (17.3 cm^2^ g^−1^), N (24.7 cm^2^ g^−1^), and H (0.4 cm^2^ g^−1^) to calculate the mass‐weighted average of the mass attenuation coefficient. This resulted in a value of 176 cm^2^ g^−1^. The density was assumed to be 4 g cm^3^. This results in information depths of 120, 232, 613, and 912 nm for incident angles 0.5°, 1°, 3°, and 5°, respectively. Prior to each measurement, the sample was carefully aligned in both height and incident angle.

Scanning electron microscopy (SEM) and Energy Dispersive X‐ray Spectroscopy (EDS) were done on a JEOL JSM‐IT700 field effect microscope. The accelerating voltage was 5 keV. The grain size distribution was obtained from the SEM images with 10kx magnifications by manually measuring the longest side of the grains using ImageJ software. Cross‐sectional SEM images were obtained by scratching a part of the film and attaching the samples to a stub with a 45° slope. The images were taken on a 10° angle with respect to the sample surface.

### Stylus Profilometry was Performed on a Veeco Dektak 8 Profilometer

X‐ray photoelectron spectroscopy (XPS) was performed on a Thermo Scientific K‐Alpha system using Al‐Kα (*λ* = 8.34 Å) radiation in an ultra‐high vacuum environment (pressure < 2 × 10^−9 ^mbar). During the measurements, the spot size was ≈800 × 400 µm^2^. The samples were loaded into the XPS from the glovebox using a vacuum transfer unit. The flood gun was operated at 0.15 mA and 1 V and the pass energy was set to 140 eV for the survey scans on the surface, and 150 eV during depth profiling. Depth profiling was achieved by use of an Ar^+^ ion beam with an energy of 2 keV with 20 s of etch time per level. The etch rate was determined by use of a reference perovskite film (thickness 275 nm) and the same etch setting. Figure S17 (Supporting Information) shows that after ≈180 s of etching, the relative contribution of oxygen, representing the silicon oxide glass substrate, significantly increases. This corresponds roughly to an etching rate of 1.5 nm s^−1^. The samples rested for 30 s after each etch level. The atomic ratios and depth profiles were determined by fitting the XPS peaks using the ThermoAdvantage software.

### Optical Characterization

Absorption spectra were recorded in ambient air using the integrated sphere on a Lambda 1050 spectrophotometer to determine the fraction of light that was either transmitted or reflected (*F*_*T* + *R*_). Subsequently, the fraction of absorbed light (*F_A_*) was calculated by equation S4.

(S4) $$F_{A}=1-F_{T+R}$$

### Photoconductance Measurements

Microwave conductivity measurements were used to probe the photoconductance of the perovskite films. This measurement relates the reduction in microwave power, caused by interactions between the microwave and mobile carriers, to the photoconductance. In the nitrogen‐filled glovebox, the samples are placed inside a resonance cavity such that a standing wave can form. This allows for the maximum overlap between the sample and the electric field component of the microwave, which greatly increases the sensitivity of the measurement. The formation of a standing wave introduces an instrumental response time of 18 ns.

Steady‐State Microwave Conductivity (SSMC) measurements are done to study the background conductivity of the samples in the dark by sweeping the microwave frequency between 8.2‐12.2 GHz. Only those frequencies that allow the formation of a standing wave result in a significant decrease in microwave power, resulting in a dip in the SSMC scan. By use of equation S5, this change in microwave power (Δ*P*) is normalized using the initial microwave power (*P*). It can be related to a (dark) conductivity by use of a sensitivity factor (*K*), a geometrical factor (β), which accounts for the inner dimensions of the cavity cell, and the sample thickness (*L*). Here, Δσ is the difference in conductivity between the sample, deposited on quartz, and a quartz reference.

(S5) $$\frac{\Delta P}{P}=-K\beta L\Delta \sigma$$

Time‐Resolved Microwave Conductivity (TRMC) measurements are employed to study the photoconductance as a function of time. Here, a pulsed laser (repetition rate: 10 Hz, wavelength: 800 nm) was used to photoexcite charges. The reduction in microwave power over time (Δ*P*(*t*)/*P*) is now related to a change in photoconductance (Δ*G*(*t*)), again using sensitivity factor K, by equation S6.

(S6) $$\frac{\Delta P\left(t\right)}{P}=-K\Delta G\left(t\right)$$

The TRMC traces can be expressed as the product of the carrier yield (φ) and the sum of the hole and electron mobilities (µ_*e*_ + µ_*h*_). As is shown in equation S7, this was calculated using the maximum change in photoconductance signal (Δ*G*_max_). This value is normalized by the fraction of absorbed light (*F*_A_) at the excitation wavelength, the intensity of the laser (*I*_0_) expressed in the number of photons per pulse per unit of area, the elementary charge (*e*) and geometry factor β.

(S7) $$\varphi \left(\mu_{e}+\mu_{h}\right)=\frac{\Delta G_{\max}}{F_{A}I_{0}e\beta }$$

### Determination of the Urbach Tail

The Urbach tail was determined by combining the absorption measurements and TRMC measurements using multiple excitation wavelengths (900, 950, 1000, 1050, 1100, 1125, and 1150 nm). The recorded photoconductance signals were due to the excitation of charges into sub‐bandgap states. The maximum photoconductance (Δ*G*_max_) was used to determine the number of photoexcited charges (*n*) using equation S8:

(S8) $$n=\frac{\Delta G_{\max}}{e\beta I_{o-R}L\Sigma \mu }$$

here, *I*_*o* − *R*_ represents the laser intensity corrected for the fraction of reflected light (*F_R_*) (equation S9), which was determined by combining absorption data recorded by placing the samples in front of the integrated sphere (*F_T_*) as well as inside (*F*_*T* + *R*_), as shown in equation S10. L is the sample thickness. The sum of the electron and hole mobilities (Σµ) can be determined from TRMC measurements using the above bandgap excitation wavelengths.

(S9) $$I_{o-R}=I_{o}*F_{R}$$

(S10) $$F_{R}=F_{T+R}-F_{T}$$

Now, the sub‐bandgap absorption coefficient (α) is determined via the transmittance (*T*), using equations S11 and S12.

(S11) $$T=\frac{I_{o-R}-nL}{I_{o-R}}$$

(S12) $$\alpha =\frac{\ln\left(T\right)}{L}$$

Next, ln (α) is plotted as a function of photon energy. Subsequently, a linear fit was applied to the data points below the bandgap energy to obtain the Urbach energy (*E_u_*) from the inverse of the slope.

Sequential thermal evaporation (sTE) can be effectively used to produce compact, structurally oriented, and crystalline low bandgap perovskite films with high photoconductance. The stacked precursor layers mix readily at room temperature, but high temperature annealing is required to obtain a favorable structure that enables high conversion efficiency and long carrier lifetimes. The addition of cesium into the A‐site of the perovskite lattice can be done successfully, yet the photoconductance of these traces is reduced compared to the formamidinium‐based perovskite. Contrary to spin‐coated samples, samples made by sTE exhibit low background conductivities, without the use of any additives.

## Conflict of Interest

The authors declare no conflict of interest.

## Author Contributions

L.P. and N.S. prepared the TE samples and performed structural, optical, and photoconductance measurements. M.M. and J.N. prepared the SC samples and performed photoconductance measurements. L.B. guided the structural measurements and analysis. L.P. analyzed the data and wrote the manuscript with guidance from R.B., A.S., and T.S.

## Supporting information



Supporting Information

## Data Availability

The data that support the findings of this study are available from the corresponding author upon reasonable request.
