# Supplementary material for: Additive‐Free Sequential Thermal Evaporation of Near‐Intrinsic Pb‐Sn Perovskites
Source: Small Methods. 2024 Dec 6;9(4):2401246. doi: 10.1002/smtd.202401246 (PMC12020349; doi:10.1002/smtd.202401246)
Supplement: Supplementary file 1 — Supporting Information [file SMTD-9-2401246-s001.docx]

Supporting Information

**Additive-free Sequential Thermal Evaporation of Near-intrinsic Pb-Sn Perovskites**

Lara M. van der Poll, Niels van Silfhout, Jasmeen Nespoli, Maartje van der Meer, Reinder Boekhoff, Lars J. Bannenberg, Arno H.M. Smets, Tom J. Savenije*


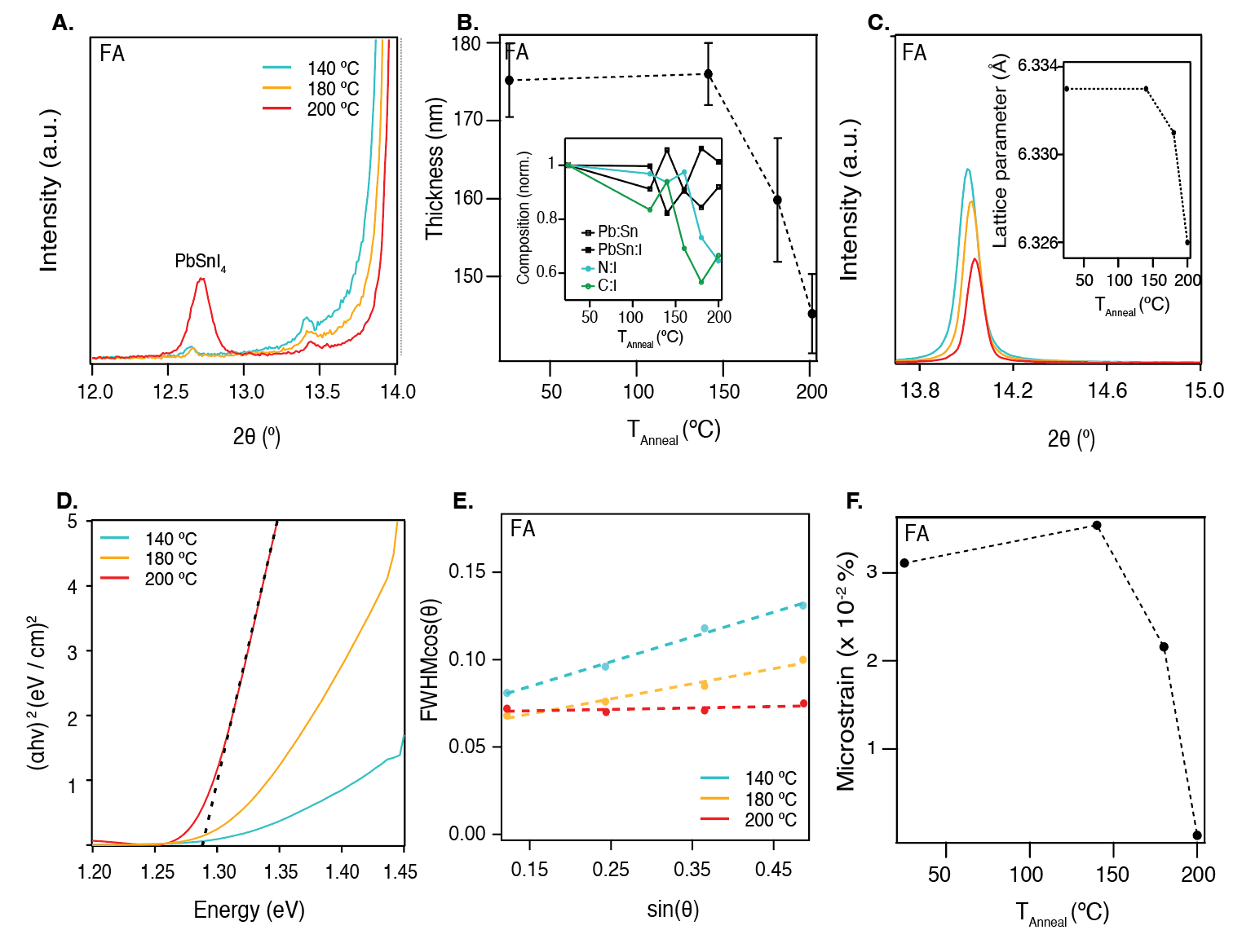


**Figure S1. A**. XRD pattern of FA annealed at 140°C**,** 180°C, and 200°C in the 2θ range of 12.0° – 14.0°. The reflection corresponding to the PbSnI_4_ alloy is labeled. **B.** Thickness of FA films as a function of annealing temperature ($T_{anneal}$). The inset shows the normalized composition as a function of $T_{anneal}$ expressed as the elemental ratios of Pb:Sn, PbSn:I, N:I and C:I. **C.** XRD pattern of FA of the samples annealed at 140°C**,** 180°C and 200°C in the 2θ range of 13.7° – 15.0°. The inset shows the lattice parameter as a function of $T_{anneal}$. **D.** Tauc plot of the FA samples with various $T_{anneal}$. The intersect of the dashed line with the x-axis represents the bandgap. **E.** Microstrain analysis of the FA samples with different $T_{anneal}$, expressed as the FWHMcosθ as a function of sinθ . The dashed lines are linear fits from which the microstrain in the (h00) direction is determined.  **F.** Microstrain as a function of $T_{anneal}$ for FA.


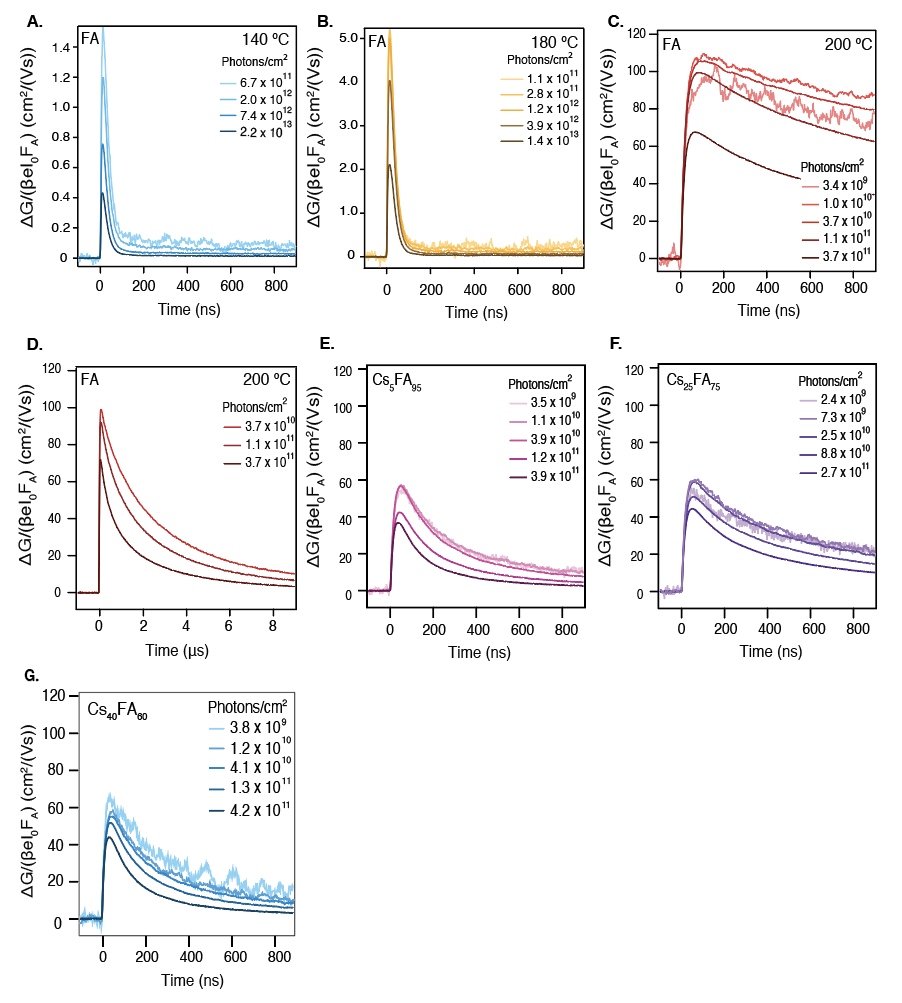


**Figure S2.** Photoconductance data as a function of time measured with laser intensities ranging between 10^9^ - 10^13^ photons / cm^2^ of FA annealed at **A**.140°C (x-scale 1μs), **B.** 180 °C (x-scale 1μs), **C.** 200 °C (x-scale 1μs), and **D.** 200 °C (x-scale 10μs). **E.** Cs_5_FA_95_ and **F.** Cs_25_FA_75_ and **G.** Cs_40_FA_60_, all annealed at 200°C, are also included. The traces are normalized by a geometric factor (β), the electronic constant ($e$), the laser intensity ($I_{0}$), and the fraction of absorbed light ($F_{A}$).


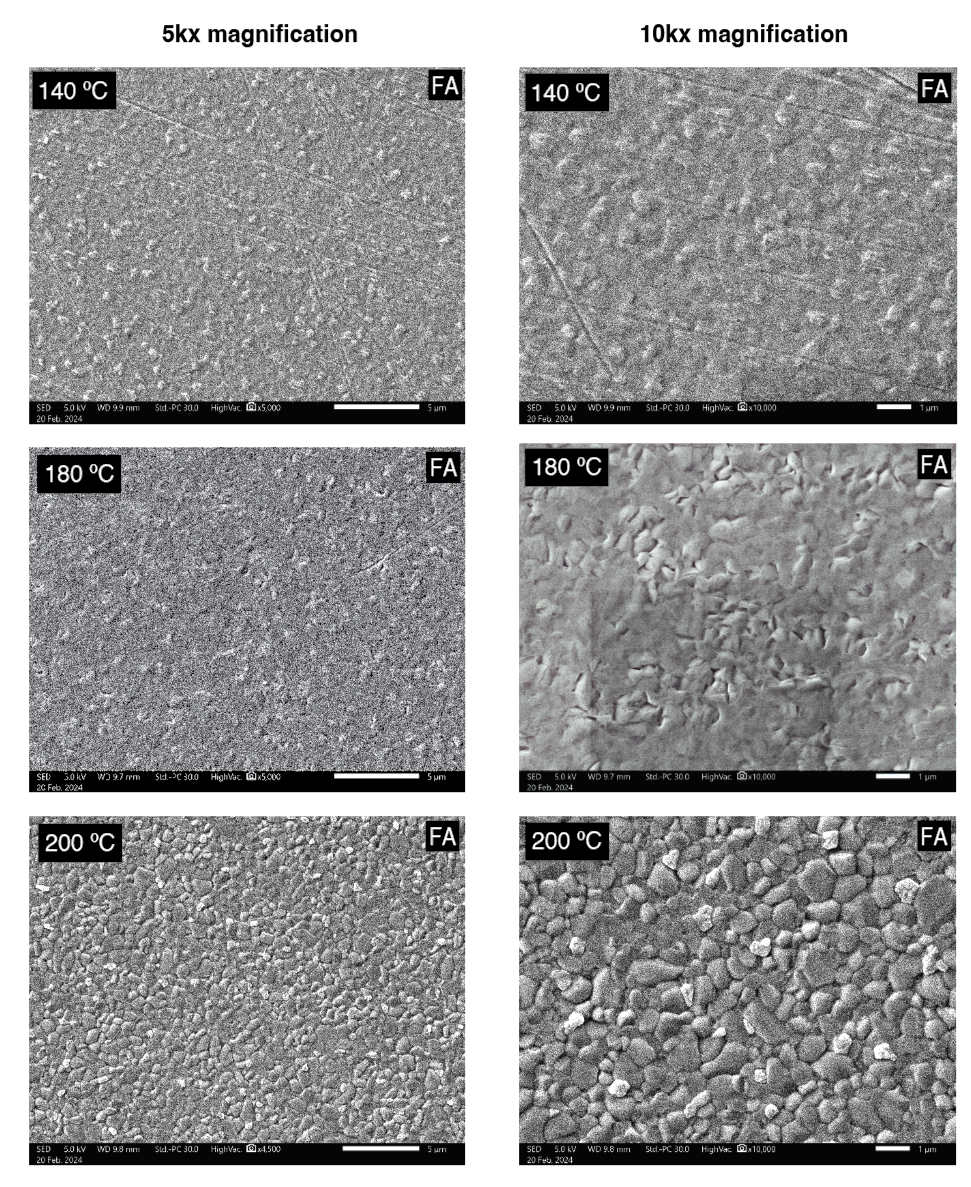


**Figure S3**. Top-view SEM images of FA samples annealed at 140 °C, 180 °C and 200 °C with 5kx (scale bar represents 5μm) and 10kx (scale bar represents 1μm) magnifications.


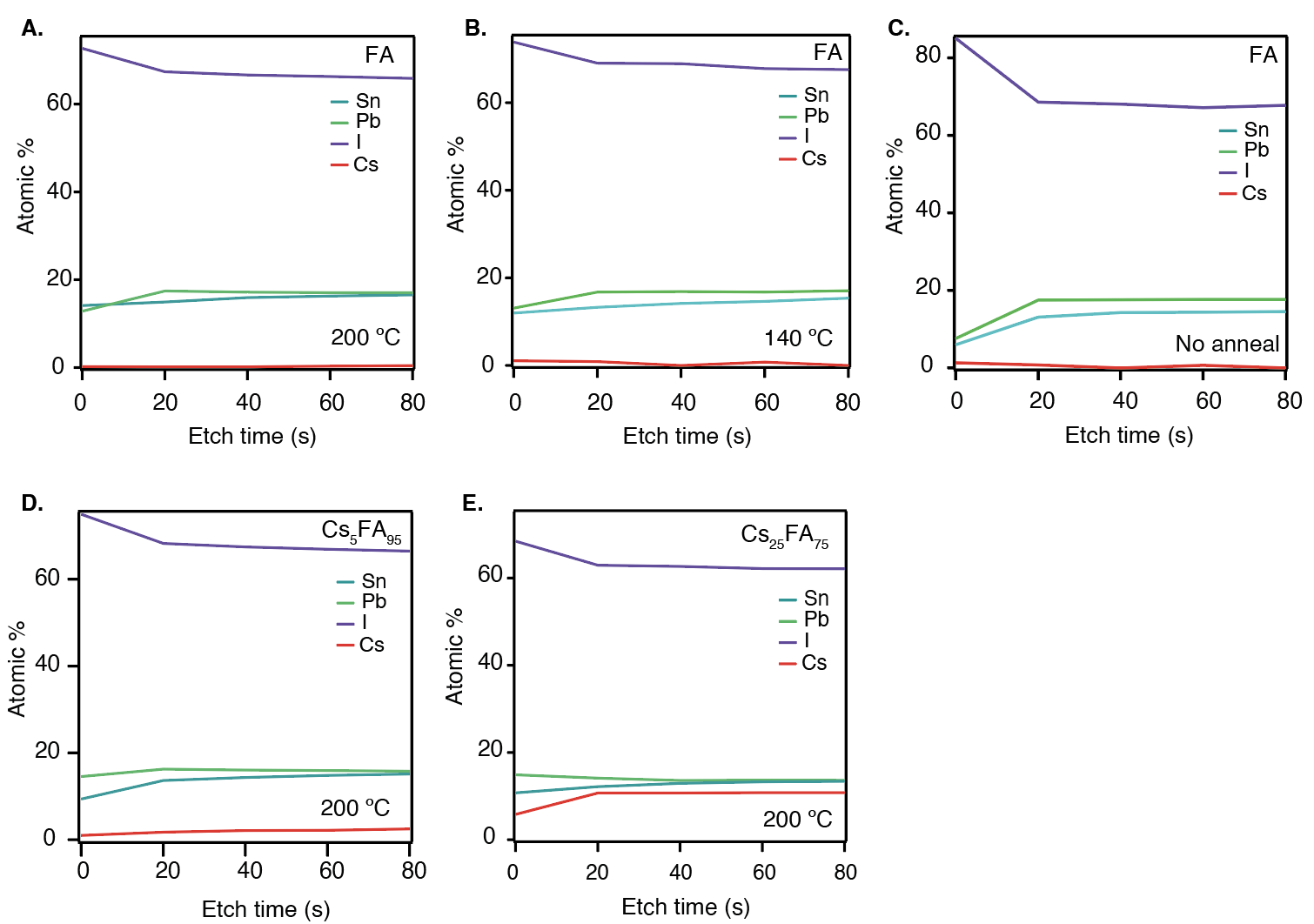


**Figure S4.** XPS depth profiles indicating the relative amounts of Sn, Pb, I, and Cs as a function of etch time of FA annealed at **A.** 200 °C, **B.** 140 °C, and **C.** FA without annealing, **D.** Cs_5_FA_95_ annealed at 200 °C, and **E.** Cs_25_FA_75_ annealed at 200 °C.


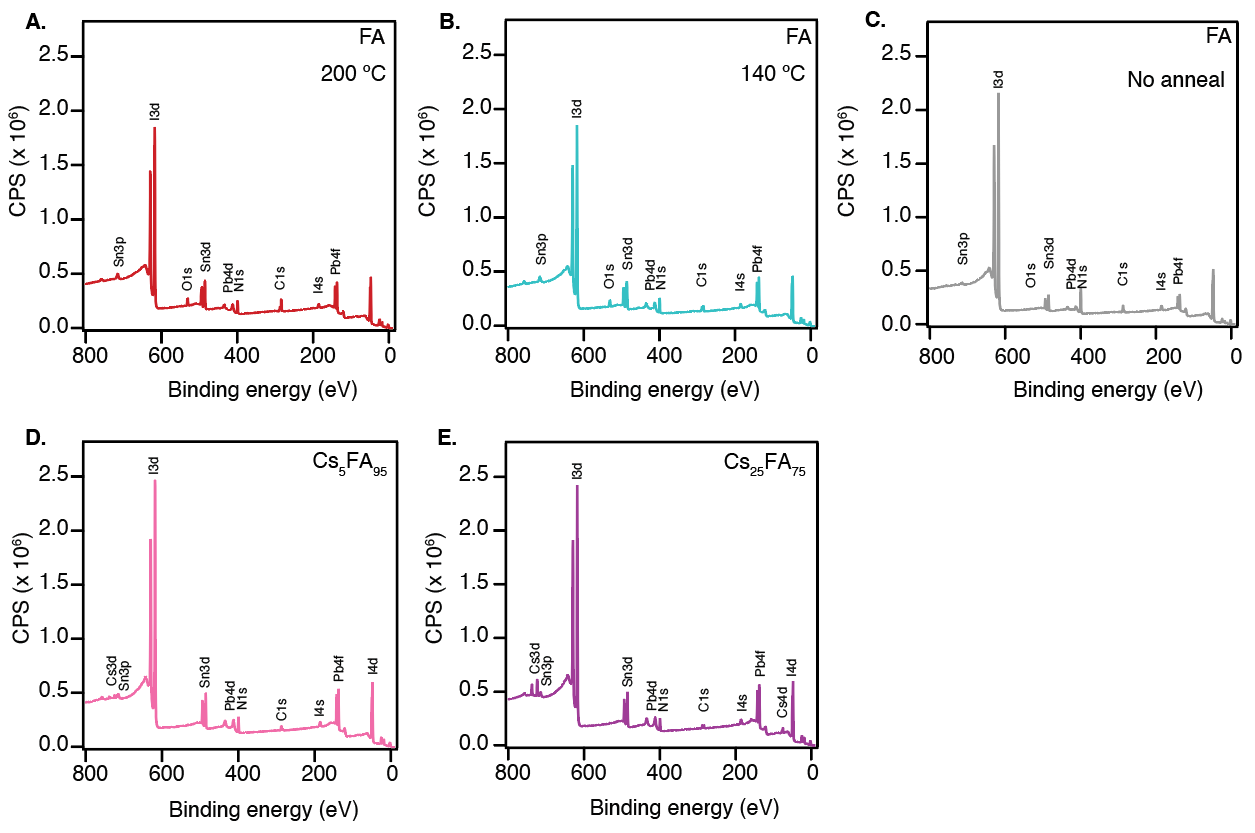


**Figure S5.** XPS survey scans of FA annealed at **A.** 200 °C, **B.** 140 °C, and **C.** FA without annealing, **D.** Cs_5_FA_95_ annealed at 200 °C, and **E.** Cs_25_FA_75_ annealed at 200 °C.


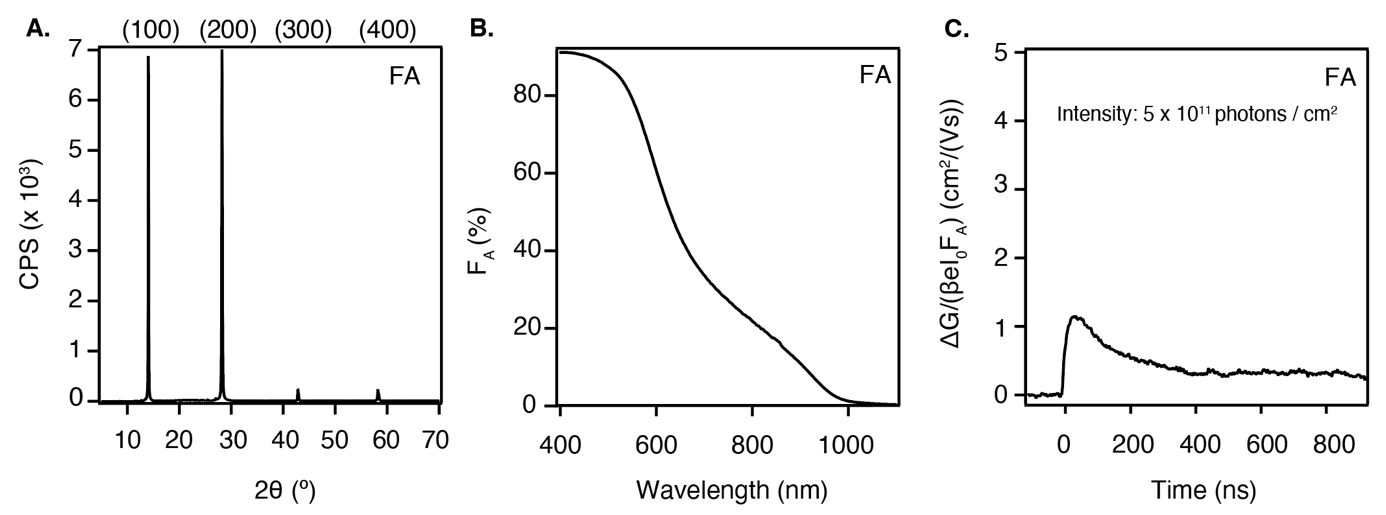


**Figure S6. A**. XRD pattern of unannealed FA film in the 2θ range of 5° – 70°. The Miller indices of the reflections are labeled above. **B.** Optical absorption spectrum of unannealed FA film expressed as F_A_ as a function of wavelength. **C.** Photoconductance data as a function of time of the unannealed FA film recorded at an intensity of 5x10^12^ photons / cm^2^. The traces are normalized by $I_{0}$ and $F_{A}$.


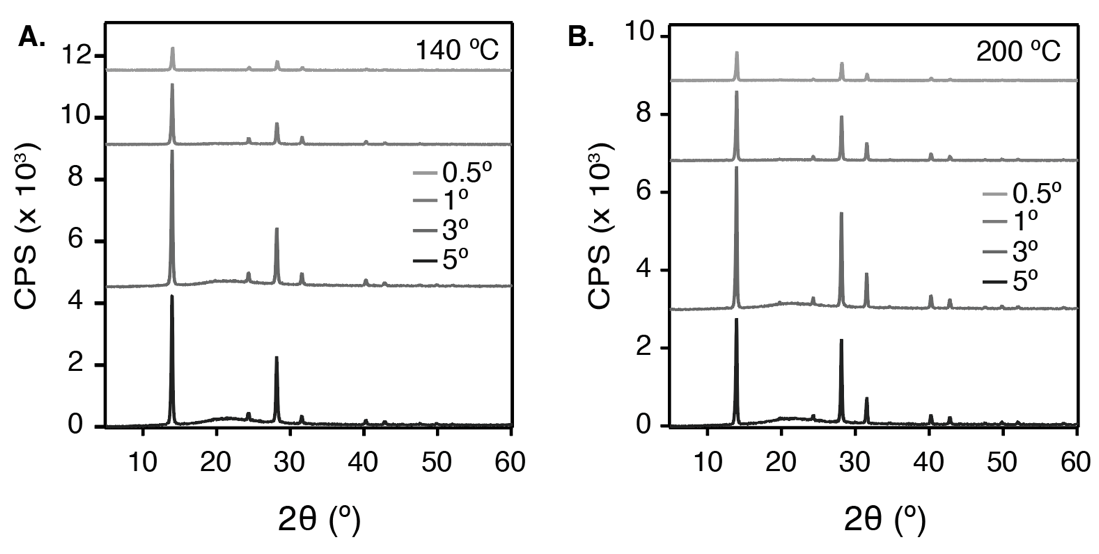


**Figure S7**. GIXRD patterns of FA annealed at **A.** 140 °C and **B.** 200 °C, recorded at incidence angles of (from top to bottom) 0.5°, 1°, 3° , and 5°.


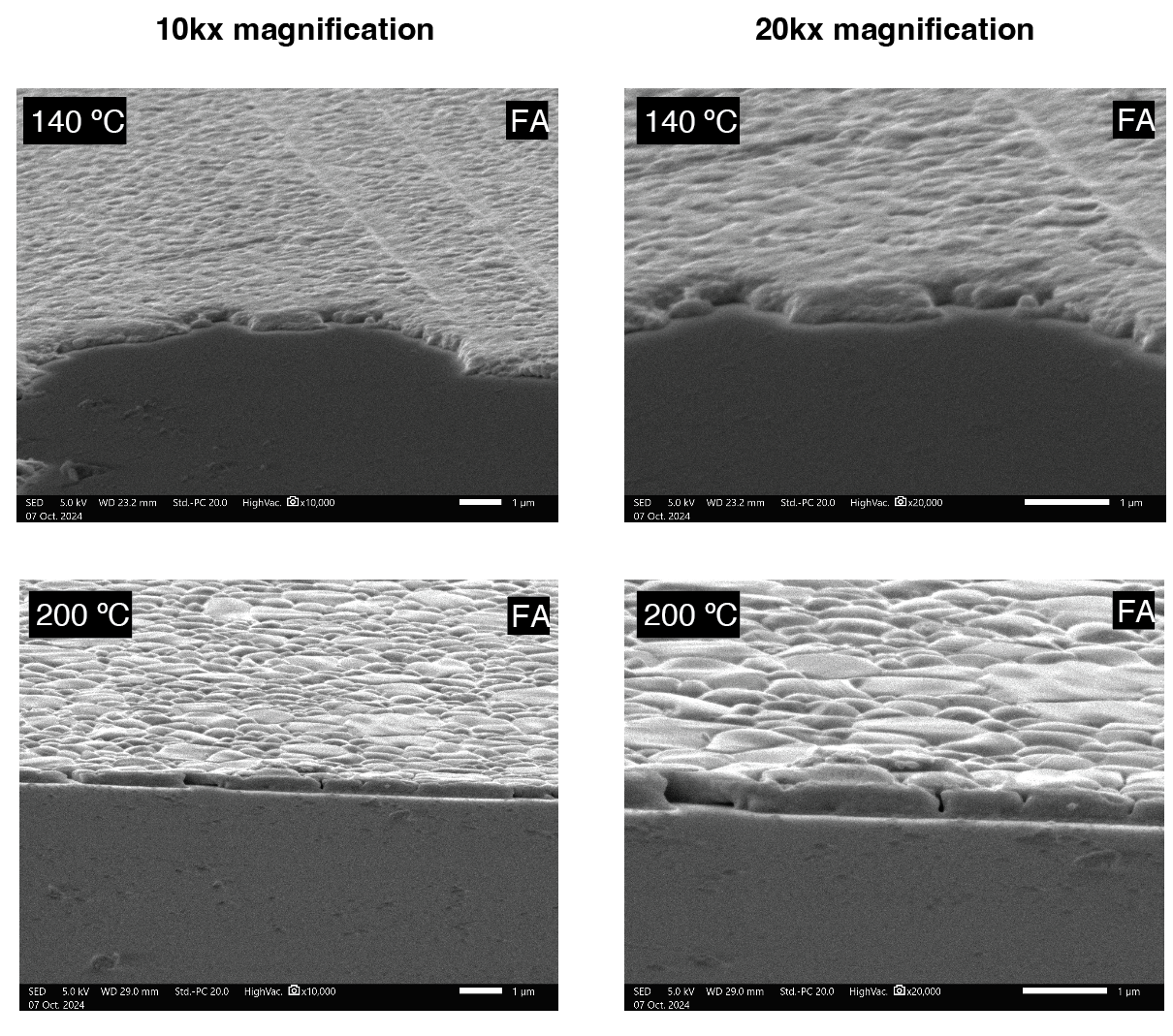


**Figure S8.** Cross-sectional SEM images of FA samples annealed at 140 °C and 200 °C with 10kx and 20kx magnifications. These were recorded at a 10° angle with respect to the sample surface. The scale bar represents 1μm.


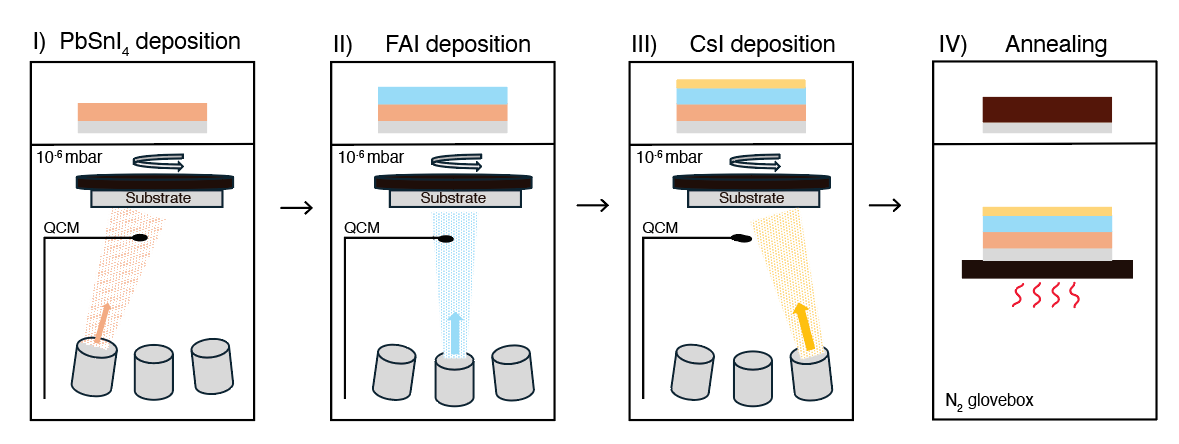


**Figure S9**. Schematic of sequential thermal evaporation of Cs_x_FA_1-x_Pb_0.5_Sn_0.5_I_3_ by I) depositing the PbSnI_3_ alloy followed by II) the deposition of FAI and III) CsI. After the deposition, the samples are transferred to the N_2_ glovebox where they are IV) annealed.

**Table S1.** Thicknesses and elemental ratios of Sn and Cs compared to Pb for FA, Cs_5_FA_95_, Cs_25_FA_75_, and Cs_40_FA_60_.

| **Sample** | **Thickness (nm)** | **Elemental ratios** | | |
| --- | --- | --- | --- | --- |
|  |  | **Pb** | **Sn** | **Cs** |
| **FA** | 145 ± 8 | 1 | 0.94 ± 0.02 | - |
| **Cs_5_FA_95_** | 147 ± 8 | 1 | 0.97 ± 0.17 | 0.13 ± 0.04 |
| **Cs_25_FA_75_** | 143 ± 5 | 1 | 0.91 ± 0.09 | 0.46 ± 0.03 |
| **Cs_40_FA_60_** | 152 ± 14 | 1 | 1.07±0.03 | 0.84 ± 0.06 |


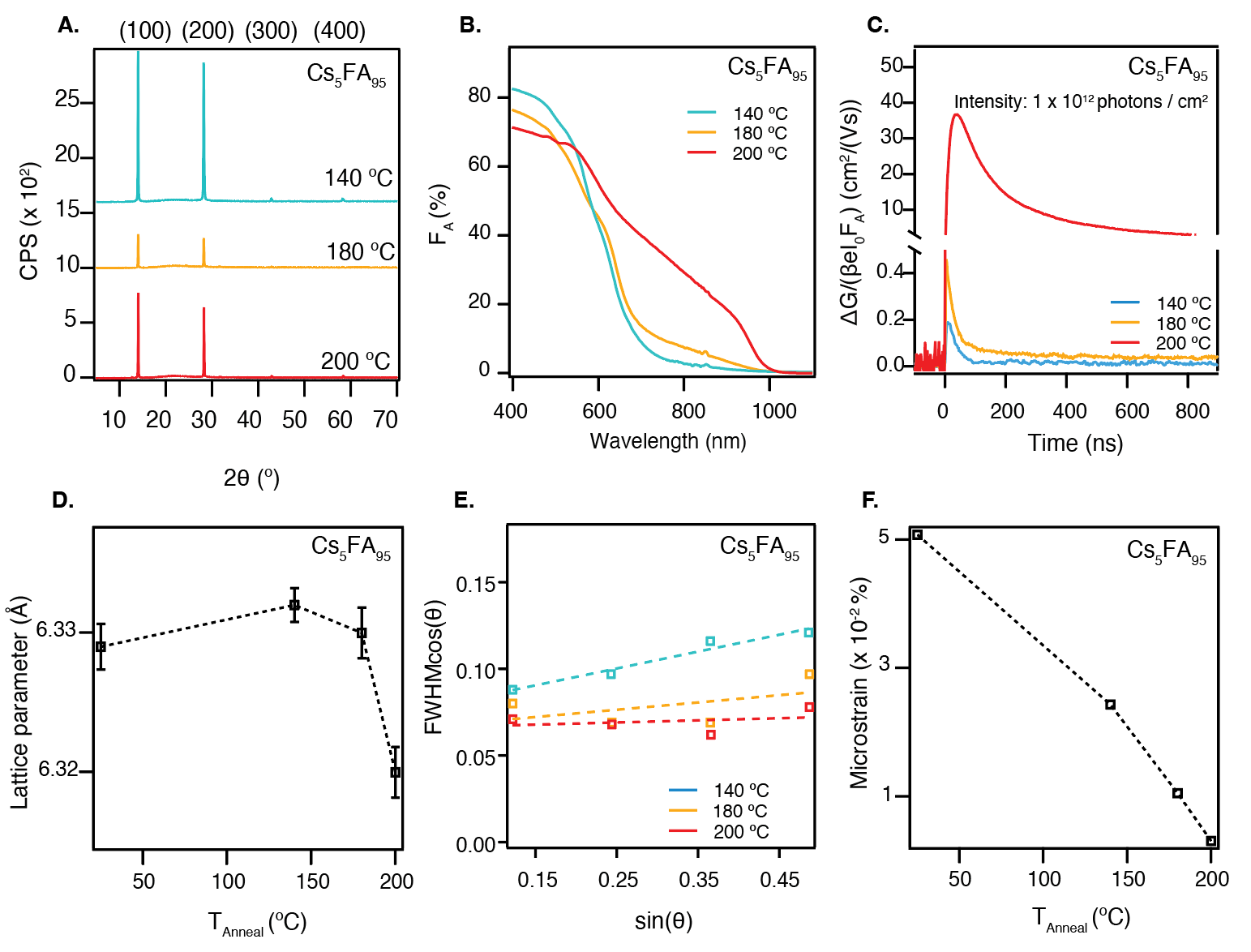


**Figure S10. A.** XRD patterns of Cs_5_FA_95_ annealed at (from top to bottom) 140°C**,** 180°C, and 200°C in the 2θ range of 5° – 70°. The Miller indices of the reflections are labeled above. **B.** Absorbance data of Cs_5_FA_95_ expressed as $F_{A}$ as a function of wavelength for the samples with various $T_{anneal}$. **C.** TRMC data of Cs_5_FA_95_ with different $T_{anneal}$ measured with 10^12^ photons / cm^2^. The traces are normalized by $I_{0}$ and $F_{A}$. **D.** Lattice parameter of Cs_5_FA_95_ as a function of $T_{anneal}$**. E.** Microstrain analysis of the Cs_5_FA_95_ samples with different $T_{anneal}$, expressed as the FWHMcosθ as a function of sinθ . The dashed lines are linear fits from which the microstrain in the (h00) direction is determined.  **F.** Microstrain as a function of $T_{anneal}$ for Cs_5_FA_95_.


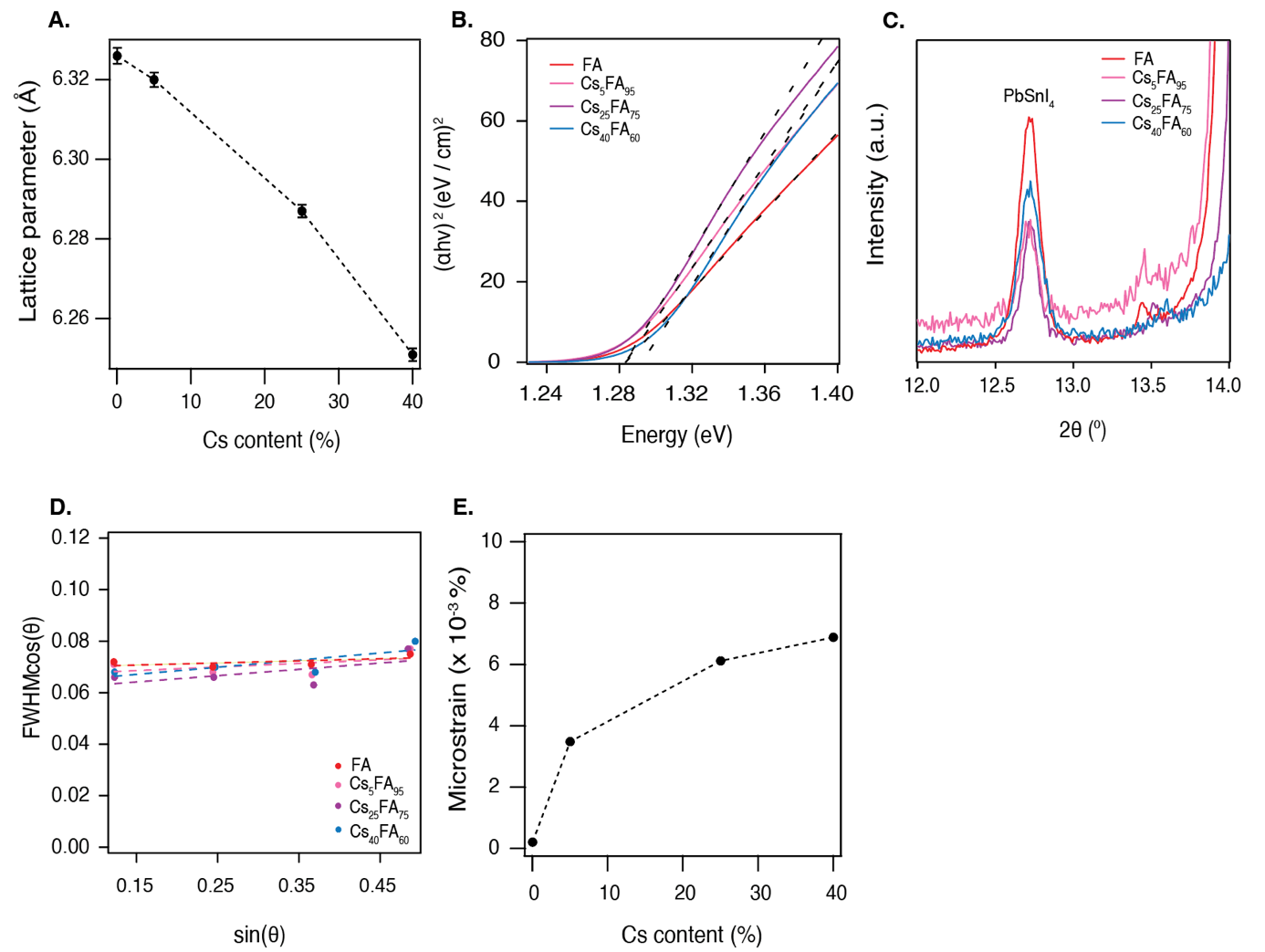


**Figure S11. A.** Lattice parameter as a function of Cs content. **B.** Tauc plot of FA, Cs_5_FA_95_, Cs_25_FA_75_, and Cs_40_FA_60_. The intersects of the dashed line with the x-axis represents the bandgap. **C.** XRD patterns of FA, Cs_5_FA_95_, Cs_25_FA_75_, and Cs_40_FA_60_ in the 2θ range of 12.0° – 14.0°. The full XRD patterns can be found in **Figure 3A**. The reflection corresponding to the PbSnI_4_ alloy is labeled. **D.** Microstrain analysis of the Cs^+^-containing samples, expressed as the FWHMcosθ as a function of sinθ . The dashed lines are linear fits from which the microstrain in the (h00) direction is determined. **F.** Microstrain as a function of Cs^+^ content.

The results presented in **Section 3** suggest that structurally, the Cs^+^-containing perovskites are similar to FA. The structural similarity between the samples is further confirmed by the response of Cs_5_FA_95_ to the same annealing series as FA. Cs_5_FA_95_ showed a similar behavior as the pure FA^+^ perovskite upon annealing, as is shown by the increase of the absorbance and the photoconductance shown in **Figure S10B** and **S10C**. Furthermore, there is a similar decrease in the lattice parameter and microstrain (**Figure S10D-F**) upon annealing. This suggests that the crystallization is similar between the compositions. To corroborate this notion, we also repeated the microstrain analysis for the remaining Cs^+^-containing samples (see **Figure S11D**). Although the microstrain slightly increases upon the addition of Cs^+^, all samples exhibit lattice parameter deviations well below 0.008% in the preferential growth direction, as shown in **Figure S11E**. Hence, we rule it out as a significant factor influencing the optoelectronic properties.


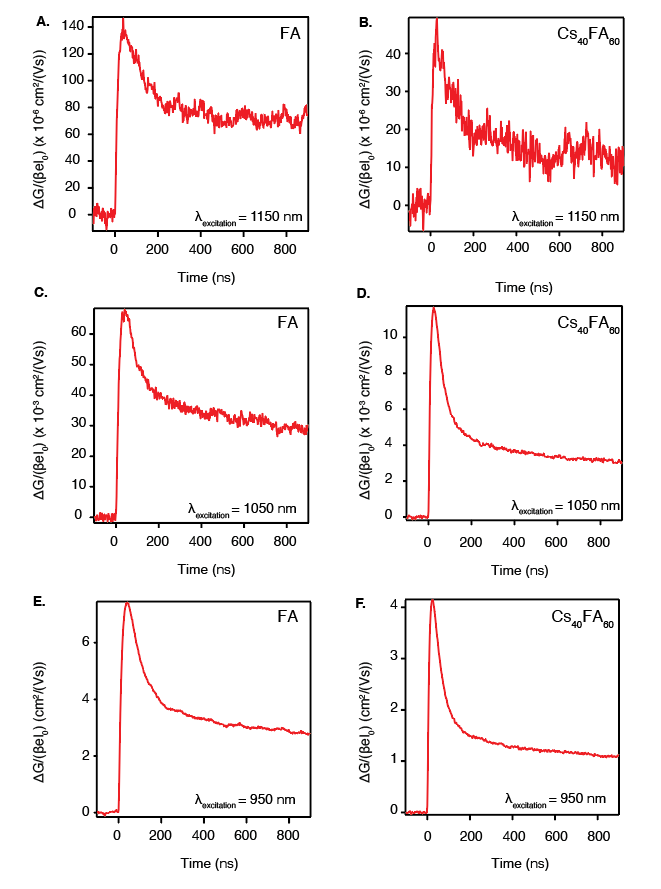


**Figure S12.** Time-resolved photoconductance data recorded at excitation wavelengths ($\lambda_{excitation}$) of 1150 nm for **A.** FA and **B.** Cs_40_FA_60_**_,_** 1050 nm for **C.** FA and **D.** Cs_40_FA_60_ and 950 nm for **E.** FA and **F.** Cs_40_FA_60_. The traces are normalized by a geometric factor (β), the electronic constant ($e$), and the laser intensity ($I_{0}$).


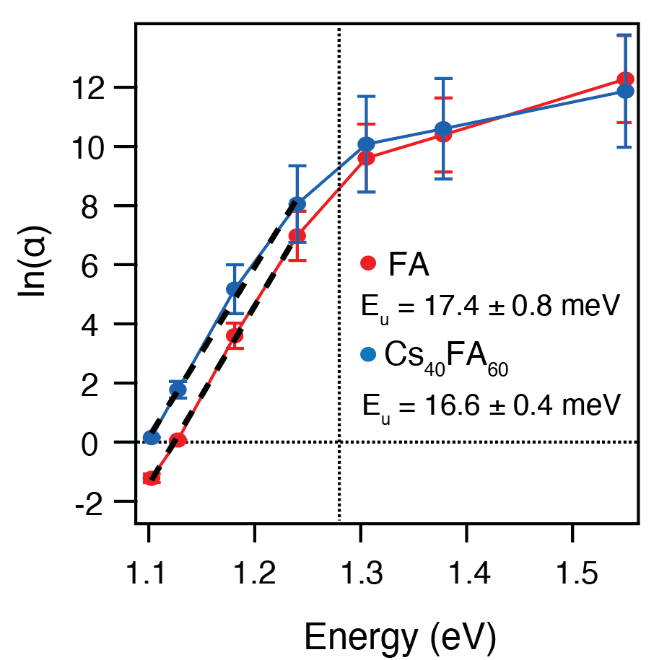


**Figure S13.** Natural logarithm of the absorption coefficient (α) as a function of photon energy for FA and Cs_40_FA_60_. α was determined by TRMC. The black dashed lines are linear fits of the absorption tail performed to determine the Urbach energy ($E_{U}$). The found values for $E_{U}$ are indicated below the key entries and are 17.4 meV and 16.6 meV for FA and Cs_40_FA_60_, respectively. The vertical black dotted line represents the bandgap energy.

The Urbach energy ($E_{U}$) represents the exponential decay of electronic states around the band edges, measured as a tail below the onset in the absorption spectrum. More disorder results in a higher $E_{U}$, which is detrimental to the cell open circuit voltage ($V_{OC}$) as it lowers the effective bandgap.^[50]^ In this work, $E_{U}$ was determined from TRMC as the high sensitivity of the cavity cell makes it possible to track the rapidly decreasing absorption below the bandgap using different intra-bandgap excitation wavelengths (see **Figure S12** for the traces recorded with excitation wavelength 950, 1050 and 1150 nm).^[51]^ The sample with the highest Cs^+^ content, Cs_40_FA_60_, is compared to FA. The results presented in **Figure S13** show that $E_{U}$ equals 17.4 ± 0.8 and 16.6 ± 0.4 meV for FA and Cs_40_FA_60_, respectively. Even though there is a slight decrease in $E_{U}$ upon Cs^+^ addition, these values are very comparable. This suggests that Cs^+^ does not affect the electronic disorder around the band edges.


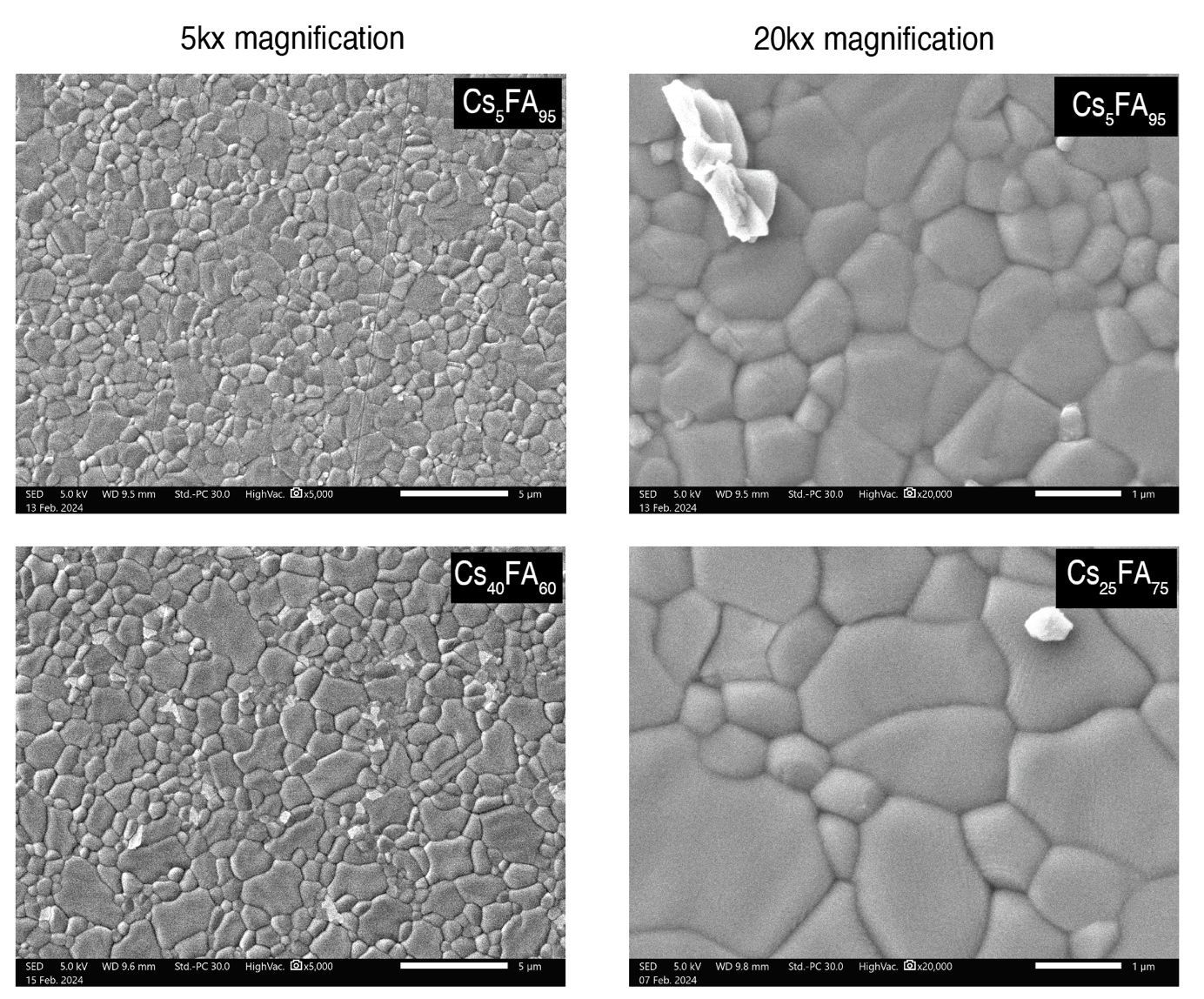


**Figure S14.** Top-view SEM images of the Cs+-containing samples with 5kx (scale bar represents 5 μm) and 20kx (scale bar represents 1 μm) magnifications.


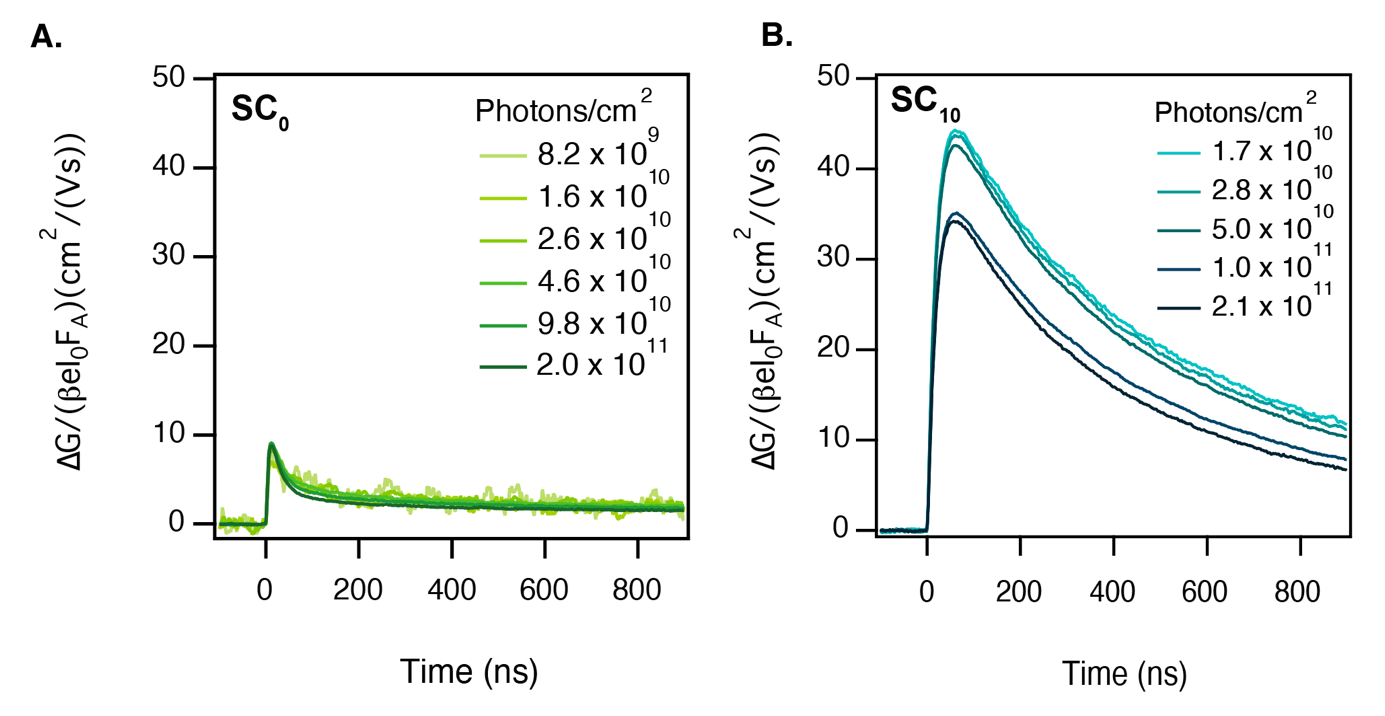


**Figure S15.** Photoconductance data as a function of time measured with laser intensities ranging between 10^11^ - 10^13^ photons / cm^2^ of SC samples with **A.** 0% SnF_2_ (SC_0_) and **B.** 10% SnF_2_ (SC_10_). The traces are normalized by a geometric factor (β), the electronic constant ($e$), the laser intensity ($I_{0}$), and the fraction of absorbed light ($F_{A}$).


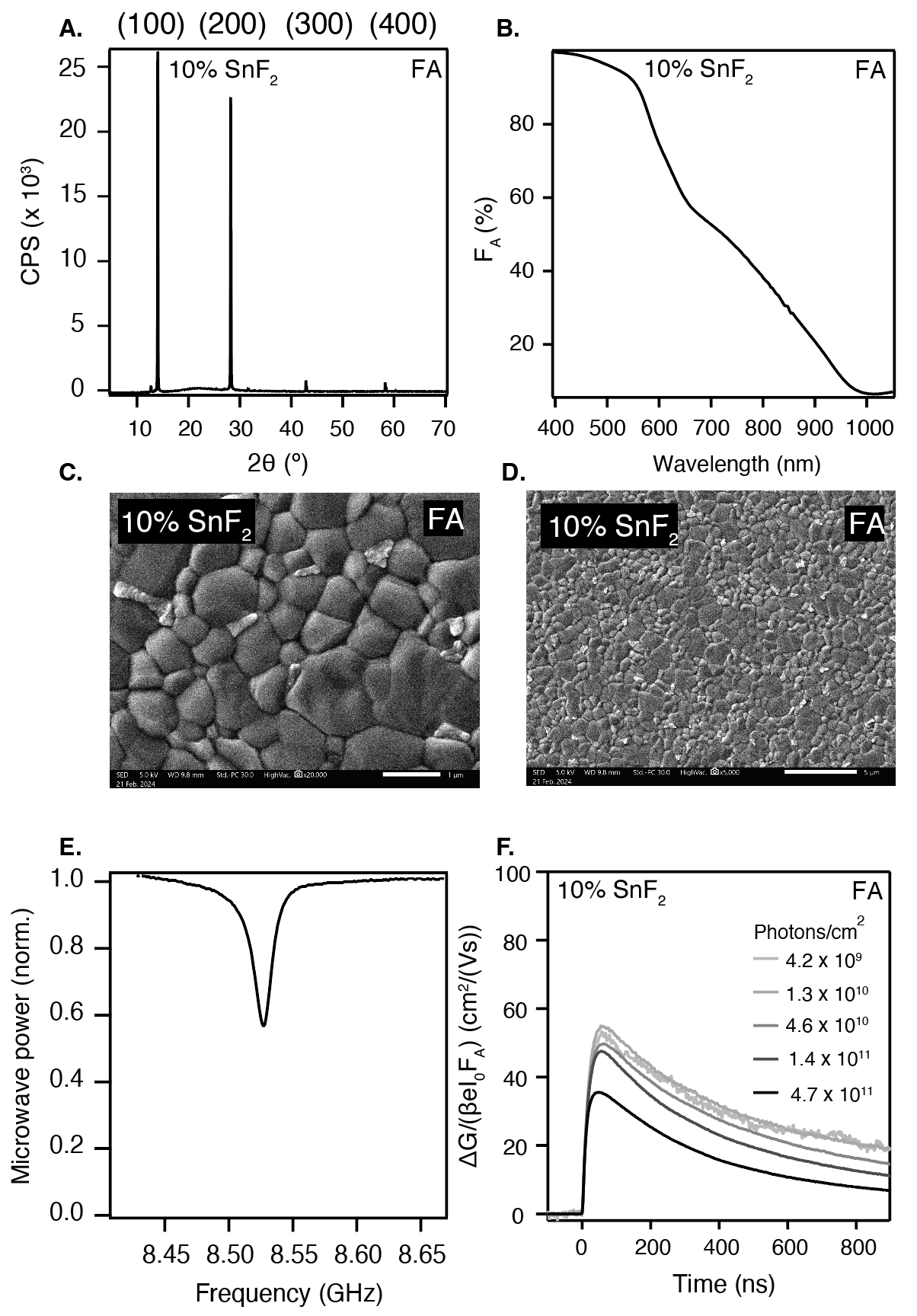


**Figure S16. A.**  XRD patterns of FA film prepared with 10% SnF_2_ added to the alloy, annealed at 200°C for 10 minutes. The Miller indices of the reflections are labeled above. **B.** Absorbance spectra, expressed as the fraction of absorbed light (F_A_ in %). Top-view SEM images of the FA prepared with 10% SnF_2_ with **C.** 20kx (the scale bar represents 1 μm ) and **D.** 10kx (the scale bar represents 5 μm) magnifications. **E.** Steady-state microwave conductivity data of FA prepared with 10% SnF_2_. **F.** Time-resolved photoconductance data measured with excitation wavelength of 800 nm. The traces are normalized by the laser intensity ($I_{0}$) and $F_{A}$.


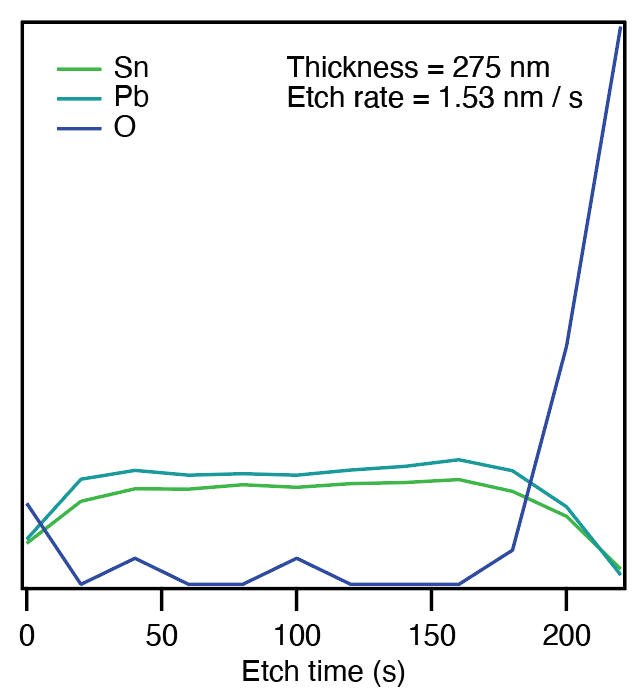


**Figure S17.** XPS depth profiling test with reference sample showing the relative contributions of Sn and Pb for the perovskite and O for the silicon oxide glass substrate.
